# The Passive Yet Successful Way of Planktonic Life: Genomic and Experimental Analysis of the Ecology of a Free-Living *Polynucleobacter* Population

**DOI:** 10.1371/journal.pone.0032772

**Published:** 2012-03-20

**Authors:** Martin W. Hahn, Thomas Scheuerl, Jitka Jezberová, Ulrike Koll, Jan Jezbera, Karel Šimek, Claudia Vannini, Giulio Petroni, Qinglong L. Wu

**Affiliations:** 1 Institute for Limnology, Austrian Academy of Sciences, Mondsee, Austria; 2 Institute of Hydrobiology, Biology Centre of the AS CR, v.v.i., České Budějovice, Czech Republic; 3 Biology Department, Protistology-Zoology Unit, University of Pisa, Pisa, Italy; 4 State Key Laboratory of Lake Science and Environment, Nanjing Institute of Geography & Limnology, Chinese Academy of Sciences, Nanjing, People's Republic of China; J. Craig Venter Institute, United States of America

## Abstract

**Background:**

The bacterial taxon *Polynucleobacter necessarius* subspecies *asymbioticus* represents a group of planktonic freshwater bacteria with cosmopolitan and ubiquitous distribution in standing freshwater habitats. These bacteria comprise <1% to 70% (on average about 20%) of total bacterioplankton cells in various freshwater habitats. The ubiquity of this taxon was recently explained by intra-taxon ecological diversification, i.e. specialization of lineages to specific environmental conditions; however, details on specific adaptations are not known. Here we investigated by means of genomic and experimental analyses the ecological adaptation of a persistent population dwelling in a small acidic pond.

**Findings:**

The investigated population (F10 lineage) contributed on average 11% to total bacterioplankton in the pond during the vegetation periods (ice-free period, usually May to November). Only a low degree of genetic diversification of the population could be revealed. These bacteria are characterized by a small genome size (2.1 Mb), a relatively small number of genes involved in transduction of environmental signals, and the lack of motility and quorum sensing. Experiments indicated that these bacteria live as chemoorganotrophs by mainly utilizing low-molecular-weight substrates derived from photooxidation of humic substances.

**Conclusions:**

Evolutionary genome streamlining resulted in a highly passive lifestyle so far only known among free-living bacteria from pelagic marine taxa dwelling in environmentally stable nutrient-poor off-shore systems. Surprisingly, such a lifestyle is also successful in a highly dynamic and nutrient-richer environment such as the water column of the investigated pond, which was undergoing complete mixis and pronounced stratification in diurnal cycles. Obviously, metabolic and ecological versatility is not a prerequisite for long-lasting establishment of abundant bacterial populations under highly dynamic environmental conditions. Caution should be exercised when generalizing the obtained insights into the ecology and adaptation of the investigated lineage to other *Polynucleobacter* lineages.

## Introduction

The current picture on diversity and function of freshwater bacteria is highly fragmentary. The most abundant taxa of freshwater bacterioplankton could be identified and, at least for some of those taxa, the respective geographic distributions and habitat preferences could tentatively be revealed (e.g. [1], [2]). However, insights into the respective ecological functions have been/were obtained only for a few taxa (e.g. [3]), and little is known about the population structure and microdiversity of abundant taxa (e.g. [4], [5]). Recent investigations have revealed quite pronounced ecological differences between seemingly closely related strains. Such strains are assigned to the same species-like freshwater taxon because they completely lack differences or differ only marginally in phylogenetic markers (i.e., ribosomal sequences), yet exhibit significant differences in habitat preferences [6], lifestyle [7] or phenotypic traits such as substrate utilization [8] and thermal adaptation [9]. These observations challenge the ecological uniformity of the lowest rank taxa (i.e. tribes) currently considered by freshwater microbiologists [2]. Conclusions from inter-habitat comparisons in particular could be biased by ecological differences between populations assigned to the same taxon. Detailed inter-habitat comparisons on diversity [6] and function of populations assigned to the same taxon are needed in order to test the comparability and generalizability of findings obtained at the currently lowest level of taxonomic rank (i.e., tribe [2]). Here we prepared the ground for extending such detailed comparisons by performing an in-depth investigation into the ecology and diversity of a single well-characterized population of *Polynucleobacter necessarius* subspecies (ssp.) *asymbioticus* [10]. The extensive knowledge of ecological function, population structure and genomic features established in this study for a particular population will enable future inter-habitat comparisons with other *Polynucleobacter* populations.

The subspecies *P.n.* ssp. *asymbioticus* (*Burkholderiaceae, Betaproteobacteria*) [10], aka PnecC bacteria [11] or PnecC tribe [2], represents a group of abundant bacteria [12] with cosmopolitan [2], [10], [11], [13], [14], [15], [16] and ubiquitous distribution in standing freshwater systems [12]. This subspecies inhabits an ecologically broad range of habitat types including small puddles, ponds and lakes with acidic, circum-neutral or alkaline conditions [1], [11], [12], [13], [17]. By contrast, to the best of our knowledge, detection of *Polynucleobacter* bacteria in marine off-shore or in soil systems has never been reported. Extrapolation of *P.n.* ssp. *asymbioticus* abundance data obtained in a systematic but geographically limited survey of more than 100 standing freshwater systems estimated an average global contribution of 20% to bacterioplankton cells in freshwater systems [12], but pronounced inter-habitat differences in relative abundance of these bacteria were observed. Contributions of up to 70% to bacterioplankton in dystrophic ponds were observed [9], [12], while in other systems cell numbers of *P.n.* ssp. *asymbioticus* were close to the detection limit [12], [18]. Both the ubiquitous distribution across different types of freshwater systems and the high average relative abundance are remarkable for a phylogenetically narrow group characterized by >99% 16S rRNA sequence similarity. However, a recent investigation employing methods further resolving the subspecies suggested that the ubiquity of the taxon resulted from ecological diversification within the taxon into lineages differing in ecological preferences and distribution across freshwater habitats (ubiquity by diversification hypothesis [6]).

It was believed that strains affiliated with subspecies *P.n.* ssp. *asymbioticus* represent chemoorganotrophs with a free-living, planktonic lifestyle [2], [10], [11], [15]. The latter was confirmed by fluorescence *in situ* hybridization (FISH) with *Polynucleobacter*-specific probes [4], [12], [17]. However, recently published findings suggest that at least some strains of the subspecies possess in addition to their chemorganotrophic metabolism a potential for phototrophy [19]. The second subspecies of *P. necessarius*, i.e. *P.n.* ssp. *necessarius*, harbors exclusively obligate symbionts of ciliates [7], [10], which implies fundamental differences in ecology and function between the two subspecies. The majority of *Polynucleobacter* strains possess small cell sizes and are classified as ultramicrobacteria (<0.1 µm^3^ cell volume) [11], [20]. Cells of such strains can pass through even 0.2 µm pore size filters [21]. Currently, in practice we lack detailed knowledge of ecological adaptation of bacteria belonging to the subspecies *P.n.* ssp. *asymbioticus* to their life in the water columns of different freshwater habitats and of lineage-specific differences in adaptation resulting in niche separation [6], [10].

The major aims of this study were (i) to reveal the ecological function of a well-characterized, persistent *P.n.* ssp. *asymbioticus* population, (ii) to obtain deeper insights in the lifestyle and population structure of the investigated bacteria, and (iii) to establish a set of reference data for future comprehensive comparisons with other populations of the subspecies. Our investigation combined a broad array of approaches: monitoring a natural population, genome analysis of one strain representing this population, phenotypic characterizations of the genome-sequenced strain, and experimental investigations with cultured and uncultured representatives of the population.

We focused on the so-called G1 (genotype 1) population [4], which represents a stable and abundant *P.n.* ssp. *asymbioticus* population in a small shallow pond (Pond-1). The G1 population forms, together with related strains known from other habitats, the so-called F10 lineage [6]. For the sake of clarity, we will use throughout this paper the more general term “F10 lineage population” for the investigated population. An overview of the current taxonomy of *Polynucleobacter* bacteria and a list of used abbreviations are provided as Table S1 and S2, respectively. The investigated F10 lineage population has been monitored from 2003 and is currently represented by a total of twelve cultivated strains obtained from Pond-1 over a period of four years. One of these strains was genome-sequenced by the DOE Joint Genome Institute, and an ecological interpretation of its genome is presented here; the description of sequencing and annotation, as well as a basic characterization of the genome, has been presented elsewhere [22].

## Results

The results section is organized into three parts. The first focuses on the environmental conditions in the pond persistently inhabited by the investigated population (F10 lineage population), as well as on the microdiversity and population structure of this population. The second part presents an ecological interpretation of the genome annotation of one strain representing the F10 lineage population as well as empirical characterizations and validations of metabolic traits of the strain. Finally, the third part presents experiments intended to further reveal and confirm the ecological function of the investigated population.

### Habitat characteristics, persistence and population structure

#### Annual and diurnal dynamics of ecological conditions in Pond-1

**Figure 1:**
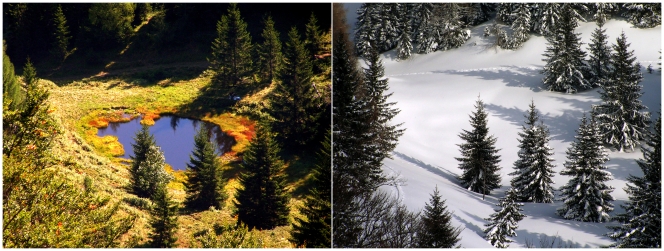
Shallow dystrophic Pond-1. This pond is persistently inhabited by the investigated *Polynucleobacter* lineage (F10 lineage). The images show the pond in autumn (left) and winter (right), when the pond is hidden below a thick ice and snow cover. The latter situation lasts for up to six months.

**Figure 2:**
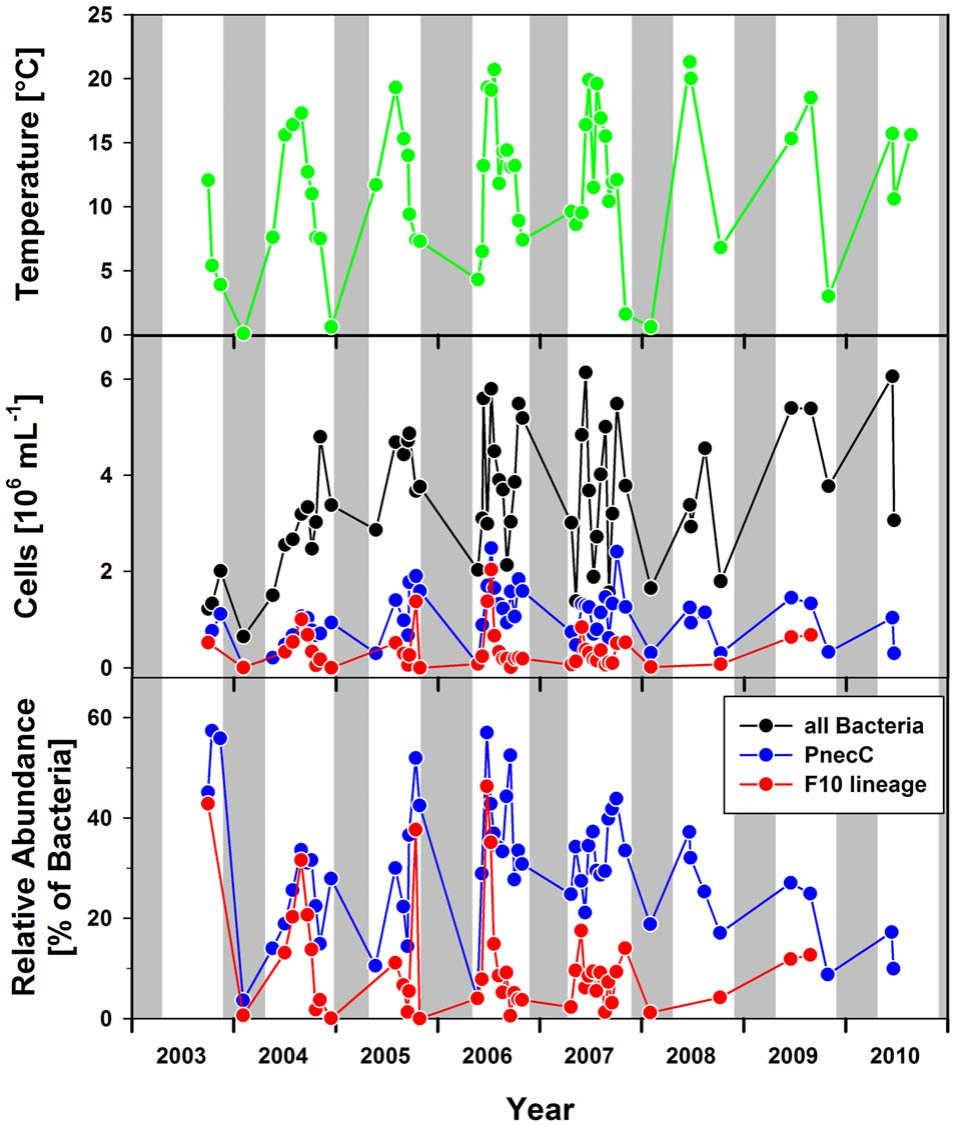
Annual cycles of water temperature and bacterial parameters of Pond-1. Water temperatures (measured in depths of about 30 cm; upper graph), abundance of the entire *P.n*. ssp. *asymbioticus* assemblage (PnecC) determined by FISH and the F10 lineage based on qPCR measurements (middle graph), and relative abundances of this two taxa (bottom graph). The gray areas indicate periods of ice-cover. Note that some samples were not investigated by qPCR.

**Figure 3:**
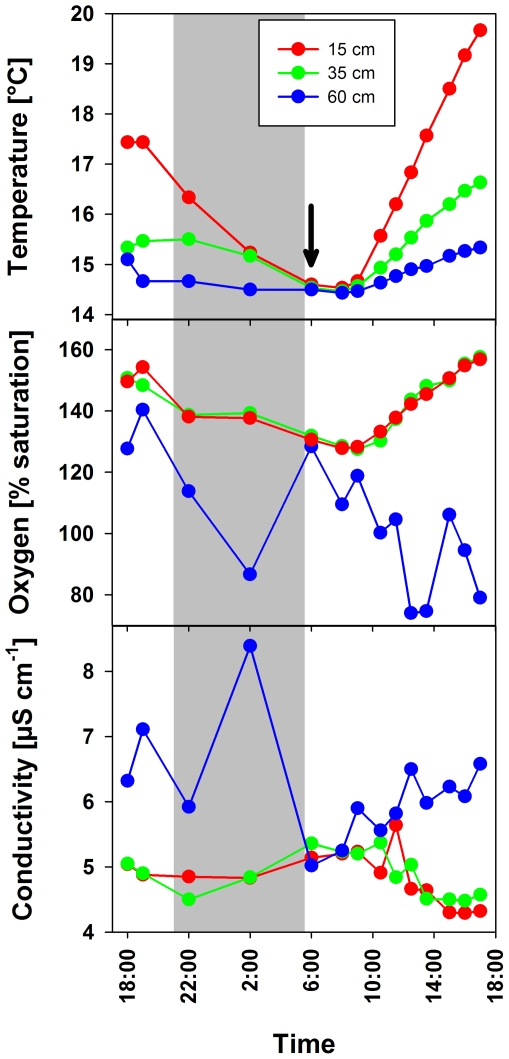
Diurnal cycles of environmental parameters in three different depths of Pond-1. Water temperature, oxygen concentration, and conductivity were measured in August 2010. The days before the recording of the diurnal cycle were characterized by cooler dull weather, while the weather conditions on the first day of measurement improved and the second day was characterized by sunny and calm weather. The arrow indicates the point in time of vertical mixing. Note that maximum temperature and oxygen values, as well as minimal conductivity values measured during the diurnal cycle were recorded in late afternoon. Since the routine sampling of the pond (Fig. 2) always took place during late-morning hours, the widths of annual variation of environmental parameters were most likely underestimated.

Pond-1 (Fig. 1) is a shallow (maximum depth of 1.2 m) small pond (surface area 200 m^2^) located (47°44′23.42″N, 13°18′6.07″E) at an altitude of 1300 m in the Austrian Alps near the city of Salzburg [4]. The habitat is seasonally ice-covered for periods of up to six months, resulting in oxygen depletion in the small water body remaining beneath the thick ice layer. The pond lacks any surface water inlet and is mainly fed by water percolating from the slopes surrounding the pond on three sides as well as by precipitation. The pond water is characterized by low ion concentrations (average conductivity 10.5 µS cm^−1^), acidic pH (range 4.3–6.0), and dissolved organic carbon (DOC) concentrations in the range of 3–9 mg L^−1^. The DOC is mainly composed of humic substances (HS). The environmental conditions in the pond show pronounced annual (Fig. 2) and even diurnal (Fig. 3) variations. Over an eight-year sampling period, water temperatures ranging from 0.1 (below ice cover) to 21.3°C and oxygen concentrations ranging from 0 (below ice cover) to almost 160% saturation were observed. During the vegetation period (ice-free period, usually May to November) the entire water column of the pond is oxic. Monitoring of environmental parameters over a 23-hour period in August 2010 revealed pronounced stratification of the water column with temperature differences of almost 5°C across a vertical distance of only 45 cm. During the night the upper water layers cooled, resulting in complete mixing of the water column by the end of the night (Fig. 3). The measured oxygen concentrations and conductivity indicated that water layers close to the bottom of the pond were influenced by the inflow of percolating water, while conditions in the upper water layers were mainly influenced by solar energy input.

In general, this habitat can be characterized as a small dystrophic polymictic pond undergoing rapid diurnal and pronounced annual changes in physical and chemical parameters. This dynamic system shares many characteristics with shallow ponds and lakes in the taiga and tundra (e.g., [13]).

#### Habitat persistence and genetic diversity of the F10 lineage population in Pond-1

**Figure 4:**
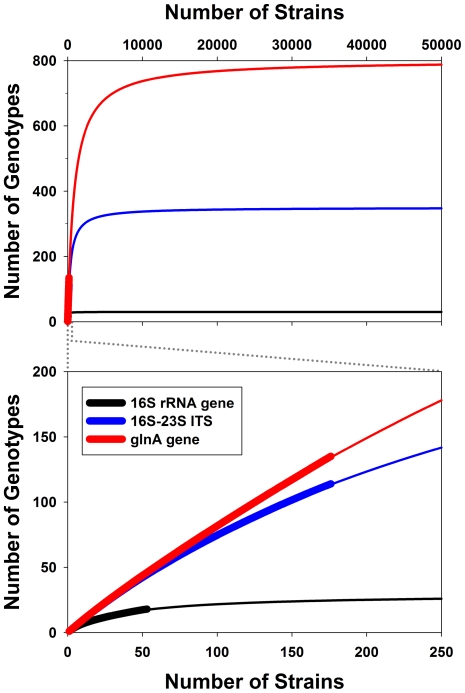
Diversity of *P. necessarius* ssp. *asymbioticus* as represented by a culture collection of 176 strains. The graphs show collector's curves depicting genotype numbers for the global non-redundant culture collection of *P.n*. ssp. *asymbioticus* strains (broad lines) after randomization of sampling order, as well as further extrapolations for continued collection of strains (thin lines). This non-redundant culture collection excludes all but one strain of the same genotype (ITS and glnA marker considered) isolated from the same habitat. Therefore, the collection includes only one (i.e. QLW-P1DMWA-1) of the twelve F10 lineage strains isolated from Pond-1 (compare Fig. 5). The collection consists currently of 176 strains isolated from various freshwater systems located all over the world. The sequencing of 16S rRNA genes of newly isolated strains was stopped after 53 strains. The length of the sequenced stretches of the markers 16S rRNA gene, 16S–23S ITS, and glnA were 1500 bp, 500 bp, and 600 bp, respectively. Note that genotype or species-richness predictions based exclusively on observed data usually underestimated the true richness. Randomization of sampling order was performed using the software EstimateS [75].

**Figure 5:**
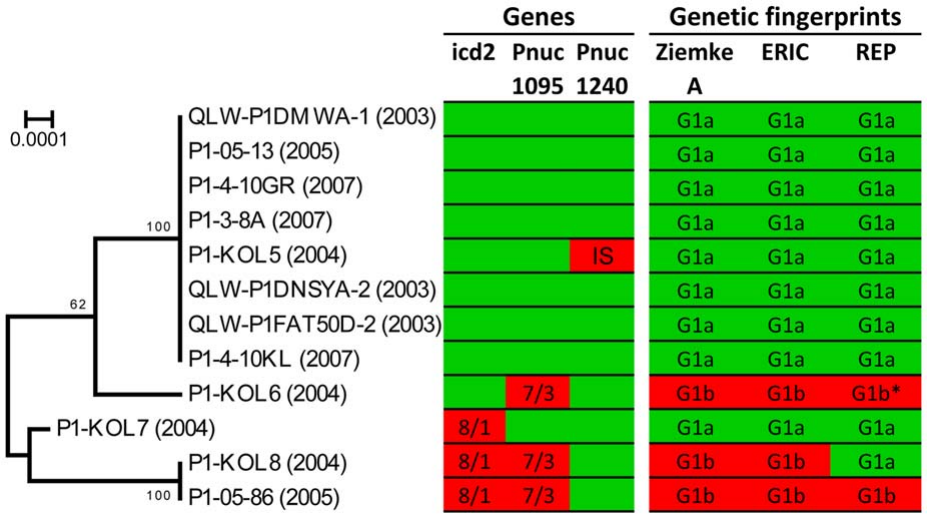
Microdiversity of the F10 lineage population of Pond-1. The genetic diversity of the twelve F10 lineage strains isolated from Pond-1 over a period of four years (2003–2007) is illustrated. **Left panel.** NJ tree based on concatenated nucleotide sequences of twelve loci (Table S4) with a total length of 9031 alignment positions. **Middle panel.** Only three out of 13 sequenced loci showed sequence polymorphism. The icd2 and Pnuc_1095 genes were present with two alleles differing in the sequenced parts in total in eight and seven positions (polymorphic positions), respectively. Numbers depict the total numbers of polymorphic sites (first number) and the number of non-synonymous sites (second number) among the polymorphic sites. The locus Pnuc_1240 of strain P1-Kol5 differed from all other sequenced strains in the presence of an insertion element (IS, insertion sequence). Sequences of Pnuc_1240 were not considered in the calculation of the presented phylogenetic tree. **Right panel.** Results from genetic fingerprinting with the independent methods RAPD (Ziemke A), ERIC, and REP-PCR. Each method resulted in different fingerprints, but each method revealed basically only two types of fingerprints (G1a and G1b). The REP-PCR fingerprint G1b* differed only weakly from G1b. Colors green and red indicate that the respective strains share or do not share, respectively, a particular trait with the genome-sequenced strain QLW-P1DMWA-1.

**Figure 6:**
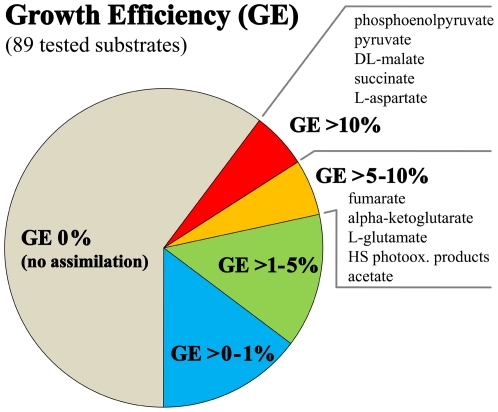
Growth efficiencies of strain QLW-P1DMWA-1 on various tested substances. Growth efficiencies (GE) of the genome-sequenced strain were determined for 89 substances by batch culture experiments. GE describes the percentage of carbon contained in the respective tested substance transferred by the strain to carbon in its biomass. Only ten substrates resulted in GE>5%. These substances are listed with decreasing efficiencies (top to bottom).

The genome-sequenced strain QLW-P1DMWA-1 and two other strains with identical 16S rRNA genes, 16S–23S ITS sequences and identical genetic fingerprints were isolated from Pond-1 in 2003 [4]. These strains were initially characterized as genotype G1 [4], which belong to the so-called F10 lineage [6]. Sequencing of two phylogenetic markers (16S–23S ITS, glutamine synthetase gene (glnA)) of a total of 176 *P. necessarius* ssp. *asymbioticus* strains indicated that the F10 lineage is genetically distinct from all other lineages within the subspecies (Fig. 4; [4], [6]; Hahn et al., unpublished data). Nine additional strains affiliated with the F10 lineage were isolated from Pond-1 in 2004, 2005, and 2007. All twelve F10 lineage strains isolated from the pond shared identical 16S–23S ITS and glnA sequences. Multilocus sequence analysis of 12 loci, as well as genetic fingerprinting by three independent methods, indicated only low genetic diversification among the twelve strains (Fig. 5). Only 15 variable positions (0.17% of all positions) were detected in the set of eleven partially sequenced genes and the completely sequenced 16S–23S ITS sequence, which represented for each strain a total sequence length of 8935 bp (0.4% of genome size). These few polymorphic sites represented single nucleotide polymorphisms located in only two genes (icd2 and Pnuc_1095) but widely spread over the sequenced parts (878 bp and 1676 bp) of these genes. Only four of the 15 polymorphic sites represented non-synonymous substitutions (Fig. 5). In the case of both genes, only two alleles were found among the twelve strains. Additionally, sequencing of gene Pnuc_1240 in all twelve strains revealed the presence of an insertion sequence element (IS) in strain P1-KOL5. Finally, genetic fingerprinting of the strains with three different methods revealed a difference between two strains (P1-KOL8 and P1-05-86) not distinguishable by the sequence data (Fig. 5). Thus, six different genotypes could be identified among the twelve strains, but the observed genetic differences are very small. No genetic differences could be revealed for seven strains (including the genome-sequenced strain) isolated in 2003, 2005, and 2007.

Besides repeated isolation of strains, application of cultivation-independent methods demonstrated the persistence of the F10 lineage in Pond-1 over the entire investigation period, i.e. 2003 to 2009 (Fig. 2). Reverse line blot hybridization (RLBH) with lineage-specific probes [6] and lineage-specific qPCR assays (Fig. 2) detected the lineage in the majority of investigated samples from Pond-1. The F10-specific RLBH assay confirmed the presence of this lineage in 41 out of 42 seasonal samples taken over the period from October 2003 to February 2008. The F10-specific qPCR assay was applied in total to 45 samples collected over a period of seven years and detected the lineage in all but one sample. *P.n.* ssp. *asymbioticus* and the F10 lineage were even detected in the ice-covered water body of the pond at mid-winter (two samplings during winter season). During the vegetation period, the F10 lineage contributed on average to 11.3% of total bacterial numbers and 35.5% of *P. necessarius* cells in the water column of the pond. However, both the relative contribution of the F10 lineage to the bacterial community and the absolute numbers of F10 lineage cells were highly variable over time but did not follow a clear seasonal pattern. In some years the F10 lineage population peaked during autumn but spring and summer peaks were also observed (Fig. 2). Thus, the investigated population did not show a clear recurrent seasonal pattern as was observed in Lake Mondsee for another *Polynucleobacter* population (or assemblage) representing another species or species assemblage (*P. acidiphobus* and/or *P. difficilis*) [23].

The pond does not receive water from tributaries but rather water that percolates from nearby slopes. Analysis of DNA extracted from water samples from a natural spring located on this slope at a distance of 350 meters by qPCR detected no *P.n.* ssp. *asymbioticus* or the F10 lineage. Consequently, the F10 lineage population can be viewed as a resident population persisting with seasonally varying population size in Pond-1 over periods of at least several years.

#### Influence of protistan predation on the P.n. ssp. asymbioticus population of Pond-1

Grazing experiments with fluorescently labeled bacteria (FLB) were conducted. Lab-grown cultures of strain QLW-P1DMWA-1 (*Polynucleobacter*-FLB) and samples of natural bacterioplankton (bacterioplankton-FLB) were fluorescently stained, and uptake of both FLB types by protistan predators present in Pond-1 was microscopically determined. At the date of the experiment, the potentially bacterivorous protistan community of Pond-1 consisted mainly of heterotrophic nanoflagellates (HNF, 3.7×10^2^ cells mL^−1^), small *Cyclidium*-like scuticociliates (3.1×10^2^ cells mL^−1^), and *Dinobryon* spp. (2.5×10^2^ cells mL^−1^). All three groups of grazers predated on *Polynucleobacter*-FLB and bacterioplankton-FLB at quite similar grazing rates. The strongest grazing impact was observed for the highly abundant *Cyclidium* sp., which consumed on average 205 bacteria cell^−1^ h^−1^, while predation rates by HNF and *Dinobryon* sp. were 8.0 and 5.1 bacteria cell^−1^ h^−1^, respectively. In total, contributions of ciliates, HNF and *Dinobryon* sp. were 93.7%, 4.4%, and 1.9% to the total bacterivory in the water column of Pond-1, respectively. Predation by these protists removed 33% of the bacterial standing stock per day, which indicated a very fast population turnover time of the target bacteria considering a rather low *in situ* temperature of 9°C. Furthermore, FISH analysis conducted directly inside flagellate food vacuoles clearly confirmed the presence of the targeted *P.n.* ssp. *asymbioticus* cells among food items ingested in food vacuoles (data not shown).

#### Growth of the *P.n.* ssp. *asymbioticus* community in Pond-1

Three processes – predation, viral lysis, and export via outflow – are expected to represent the major loss factors of the *Polynucleobacter* community in Pond-1. These losses have to be compensated by growth since an import of *Polynucleobacter* cells from the terrestrial environment is highly unlikely. Only loss by protistan predation has been quantified so far. Based on the estimated grazing mortality of *Polynucleobacter* bacteria of 33% of cells per day, a minimum growth rate of these bacteria in the same order of magnitude could be expected.

### Phenotypic and genomic traits of strain QLW-P1DMWA-1

#### Substrate assimilation tests

In total, 93 substances were empirically tested for assimilation by pure cultures of strain QLW-P1DMWA-1 (Table S3). Sixty percent of the substances did not support growth of the strain, and among the substances yielding growth only assimilation of ten (11% of total) resulted in efficiencies >5% (Fig. 6). Nine out of these ten substances were carboxylic acids with two to five carbon atoms. Five of these compounds represent intermediates of the central metabolism, especially of the tricarboxylic acid cycle (TCA). Furthermore, two amino acids and one typical fermentation product were included. In addition, the efficiency for growth on photooxidation products of humic substances (HS) was estimated to be 9%. However, no significant growth was observed for untreated HS (see below). Interestingly, no growth or only weak growth was observed for all tested carbohydrates, as well as for most of the amino acids.

#### Nitrogen and sulfur metabolism

**Figure 7:**
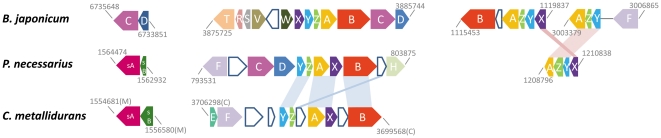
Comparison of SOX gene clusters. The SOX gene clusters of strain QLW-P1DMWA-1 (*Burkholderiaceae*, *Betaproteobacteria*), *Bradyrhizobium japonicum* (*Alphaproteobacteria*) and *Cupriavidus metallidurans* (*Burkholderiaceae*, *Betaproteobacteria*) are compared. The three clusters of the *Polynucleobacter* strain are represented by the genes Pnuc_1486, Pnuc_1487, and Pnuc_0799 - Pnuc_0809, and Pnuc_1154 - Pnuc_1157. Start- and end-positions of the clusters in the respective genome sequences are depicted. Shaded areas indicate if *Polynucleobacter* genes are closer related to *Cupriavidus* or *Bradyrhizobium* genes.

The genome of QLW-P1DMWA-1 encoded a conserved cluster of 13 genes (Pnuc_1190 - Pnuc_1202) also found in *Ralstonia*, *Cupriavidus* and *Burkholderia* strains. This cluster encoded a putative ABC transporter for urea, a urease consisting of three subunits as well as some accessory genes related to urease activity. Growth with urea as sole nitrogen source could be observed in experiments with acetate as sole energy and carbon source. A region of 16 genes (Pnuc_0987 - Pnuc_1003) encoded genes predominantly involved in nitrogen assimilation. This includes putative transporters for amino acids and nitrate, a cyanate transporter and a cyanate hydratase, as well as reductases for assimilation of nitrate and nitrite. This region consists of conserved modules of three or four genes, which are also found in other *Betaproteobacteria* or other *Proteobacteria*. Growth experiments revealed that strain QLW-P1DMWA-1 is able to grow with combinations of nitrate and sulfate, nitrate and thiosulfate, urea and sulfate, urea and thiosulfate as sole nitrogen and sulfur sources, respectively. Pnuc_1350 encoding a putative rhodanase could be responsible for the capability to use thiosulfate as sole sulfur source. Furthermore, a region of the genome containing genes Pnuc_1476 to Pnuc_1494 encodes several functions involved in assimilation of inorganic sulfur. The presence of SOX genes (Fig. 7) suggested that the strain is able to gain energy from oxidization of inorganic sulfur species such as H_2_S. However, this trait has not yet been experimentally confirmed.

#### Genome characteristics and genome evolution

**Figure 8:**
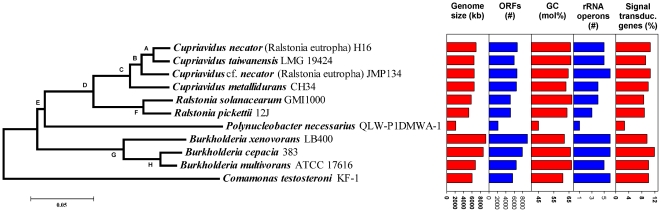
Phylogenetic position of the genome-sequenced *Polynucleobacter* strain. Reconstruction of the phylogenetic position of the genome-sequenced strain *P.n.* ssp. *asymbioticus* QLW-P1DMWA-1 and genome characteristics of the strains used for the phylogenetic analysis. The presented tree is based on concatenated alignments of amino acid sequences of proteins encoded by eight housekeeping genes and was constructed using the NJ method. Letters A to H refer to bootstrap values obtained for the respective nodes by three different treeing methods in analyses of nucleotide and amino acid sequence data sets. The bootstrap values are presented in Table S7.

**Figure 9:**
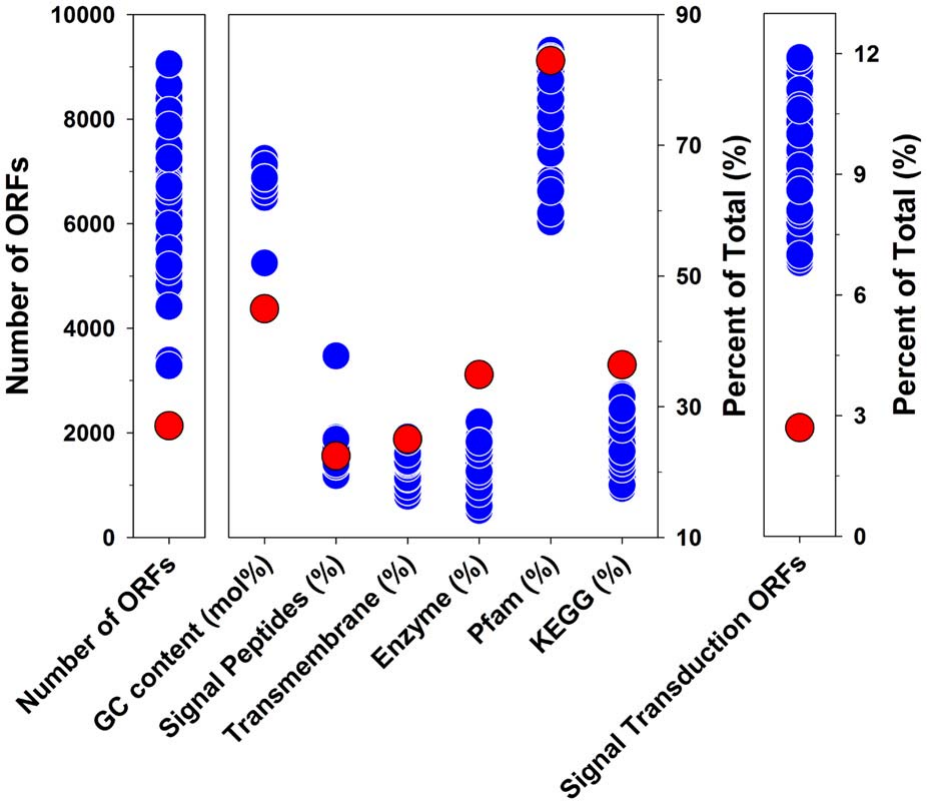
Variability of genomic traits among genome-sequenced members of the family *Burkholderiaceae*. The depicted traits are the total number of ORFs, G+C content of the genomes, percentage of CDS encoding signal peptides, transmembrane domains, genes assigned to Enzyme Commission (EC) number classes, proteins assigned to protein families (Pfam), proteins assigned to KEGG categories, and proteins involved in signal transduction. For most traits, 78 genomes of strains affiliated with the family were analyzed using the IMG system [24]. The sole exception is the analysis of signal transduction genes, which was performed using the MIST2 database [69], which provided data on 37 strains affiliated with the *Burkholderiaceae*. For all depicted parameters, all available genomes of family members were considered but not the genome of the endosymbiotic *Polynucleobacter* strain STIR1. Data of strain QLW-P1DMWA-1 are indicated as red dots.

**Figure 10:**
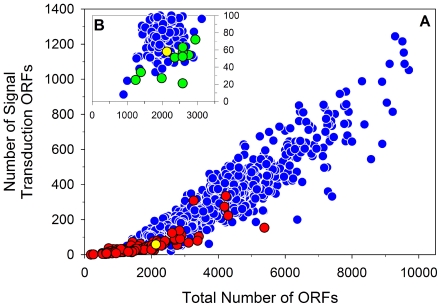
Gene number of bacterial genomes versus number of encoded signal transduction genes. The plot depicts the total number of annotated signal transduction genes in particular bacterial genomes versus the total number of genes of the respective genomes. Data on *Archaea* are not shown, and multiple genomes per species or species-like taxa were excluded. Strain *P. necessarius* QLW-P1DMWA-1 is highlighted as a yellow dot. (A) Entire data set consisting of 904 analyzed bacterial genomes. Red dots indicate bacteria obligately associated as symbionts or pathogens with a host; blue dots indicate free-living or facultative host-associated bacteria. (B) Plot of genomes of bacteria classified as free-living or facultative host-associated (shown in (A) as blue dots) with ≤100 signal transduction genes. All blue dots represent bacteria either facultatively associated with hosts (e.g., dwelling in the oral cavity or in the intestinal tract) or dwelling as free-living organisms in extreme environments (permanently anoxic, low or high temperature, low pH). The yellow (strain QLW-P1DMWA-1) and the green dots represent the only exclusively free-living taxa inhabiting non-extreme systems. Almost all of them are phototrophic (*Synechococcus* and *Prochlorococcus*) or predominantly heterotrophic taxa (*Pelagibacter* and *Puniceispirillum*) dwelling as planktonic bacteria in marine systems. The signal transduction gene data were obtained from the database MIST2 [69].

A phylogenetic analysis based on multilocus sequences of protein-encoding housekeeping genes (Fig. 8) confirmed previous phylogenetic analyses solely based on 16S rRNA gene sequences [10], [11]. Accordingly, the genus *Polynucleobacter* is affiliated with the family *Burkholderiaceae*, and the closest related described genera are *Ralstonia* and *Cupriavidus*. The genus *Polynucleobacter* is currently the sole taxon within the family *Burkholderiaceae*, which contains bacteria with a planktonic lifestyle. All related taxa either represent free-living soil bacteria or bacteria associated with plants or animals. The 2.15 Mb genome of strain QLW-P1DMWA-1 consists of a single replicon (Table 1) and is significantly smaller than the genomes of other currently sequenced strains belonging to the *Burkholderiaceae* (Fig. 9). Genome sequences of 78 strains affiliated to this family are currently available in the Integrated Microbial Genomes (IMG) system [24]. The size range (excluding two *Polynucleobacter* genomes) of these genomes is 3.3 to 9.7 Mb, and the average size (without *Polynucleobacter*) is 6.9 Mb. Furthermore, the genome of the strain differs remarkably in several parameters including, for example, G+C content of DNA (Fig. 9). Interestingly, the genome encoded unusually high percentages of genes that could be classified in Enzyme Commission (EC) number categories or assigned to one of the Kyoto Encyclopedia of Genes and Genomes (KEGG) categories (Fig. 9). The genome was characterized by the lack of many genes present in related organisms (see Text S1). The ecologically most interesting traits lacking were motility and photosynthesis (compare reference [19]). Importantly, the genome of QLW-P1DMWA-1, for a free-living strain, encoded an unusually low absolute and relative number of signal transduction genes (Figs. 8, 9, 10). This is a feature shared with probably all marine planktonic bacteria with streamlined genomes, e.g. SAR11 bacteria (including *Candidatus* Pelagibacter ubique) and *Prochlorococcus* spp. (Fig. 10B). The genome annotation revealed no reliable indications of an adaptation to a host-associated lifestyle.

**Table 1:** Basic features characterizing the genome of *P. necessarius* ssp. *asymbioticus* strain QLW-P1DMWA-1.

Feature	QLW-P1DMWA-1
Genome size (Mb)	2.16
G+C content (mol %)	44.8
Number of replicons	1
Extrachromosomal elements	0
Coding region (% of bp)	93.1
Total genes	2136
Protein-coding genes	2088
rRNA operons	1

For 278 (13.3%) of the protein-encoding ORFs of the genome, analysis employing the IMG system indicated a putative origin by horizontal gene transfer. The majority of these genes (74.2%) were putatively obtained from other *Proteobacteria*. The three classes *Alphaproteobacteria* (22.9%), *Betaproteobacteria* (20.8%), and *Gammaproteobacteria* (21.1%) seem to have contributed about equally to the total numbers of horizontally acquired genes. For example, the genome contained genes involved in oxidization of inorganic sulfur (SOX genes, Fig. 7). Sulfur oxidization pathways are also present in *Cupriavidus* and *Ralstonia* strains (e.g. [25]). However, the genome of QLW-P1DMWA-1 contained particular SOX genes not present in those taxa but in *Alphaproteobacteria* such as *Bradyrhizobium japonicum* [26].

Despite the pronounced differences in ecological adaptation and habitat preferences between QLW-P1DMWA-1 and other genome-sequenced members of the family *Burkholderiaceae*, genes characterized by both a beneficial role in adaptation to a planktonic lifestyle and absence in other *Burkholderiaceae* remain hard to identify. A more detailed analysis of this topic is presented in the Text S2.

#### Metabolic reconstruction

The genome of strain QLW-P1DMWA-1 (F10 lineage) encoded genes for a complete tricarboxylic acid cycle, Embden-Meyerhof-Parnas pathway (glycolysis), a complete glyoxylate cycle, and encoded an acetyl-CoA synthetase enabling utilization of acetate. The glycolysis and gluconeogenesis pathways seemed to be completely encoded by the genome, however, genes encoding kinases for phosphorylation of α- or β-D-glucose seemed to be lacking. Since kinases for other hexoses as well as a phosphotransferase system (PTS) were also missing, it seems to be unlikely that the bacterium is utilizing this pathway for degradation of hexoses. The strain lacked the oxidative part of the pentose phosphate pathway and an Entner-Doudoroff pathway. Genes of pathways for *de novo* synthesis of all but one common cofactor and vitamins were found in the genome. In several cases the majority of genes required for a complete biosynthesis pathway were annotated, but one or two genes seemed to be lacking (e.g. thiamine and biotin pathways). The only fully absent pathway was the one for biosynthesis of vitamin B12. Since the genome of the strain encoded at least two B12-binding enzymes (i.e. Methylmalonyl-CoA mutase (Pnuc_0910), methionine synthase (Pnuc_1979)), this vitamin is most likely acquired by using transporters. Furthermore, genes for *de novo* synthesis of all twenty common proteinogenic amino acids apart from selenocystein could be identified in the genome. A tRNA gene for selenocystein is also lacking, while at least one tRNA gene for each common amino acid is encoded. Previously demonstrated aerobic growth of strain QLW-P1DMWA-1 on acetate as sole carbon and energy source in an inorganic medium exclusively supplemented with vitamin B12 [10] suggested that all other vitamins and cofactors, as well as amino acids and other substances required for growth, can be synthesized from precursors provided by the TCA.

In previous investigations, anaerobic growth of strain QLW-P1DMWA-1 could be observed [10]. The genome annotation revealed no reliable indications of the kind of anaerobic metabolism performed by the strain. Nitrate respiration seemed unsupported by the annotation. Further investigations must reveal if anaerobic growth results from anaerobic respiration or from fermentation. However, survival or growth under anoxic conditions is expected to be of ecological relevance due to the lack of oxygen in the water body of the pond under longer-lasting ice-covered conditions (Figs. 1 and 2).

Analysis by TransportDB resulted in no indications of specific putative substrates for the majority of putative transporters. However, based on the analysis, many putative transporters could be assigned to functional groups. These assignments indicated that only a small number (three genes, 1.4% of genes putatively involved in transport) could be related to ‘import of carbohydrates’, whereas in total 17.7% of transporter proteins were assigned to the two categories ‘import of carboxylic acids’ (4.5%) and ‘import of amino acids’ (13.2%). Twenty-five and 36.4% of the transporter genes were assigned to the ‘import of inorganic nutrients’ and ‘export and efflux’ categories, respectively. The assignment of the three genes to the ‘import of carbohydrates’ category could be misleading since one assignment is based only on a low E-value (Pnuc_1331) and one other assignments represented a protein (major facilitator family transporter) putatively involved in transport of a broader spectrum of substrates including sugar phosphates, tartrate and phthalate (Pnuc_1635). Actually, the genome seemed to be lacking any ABC transporters for carbohydrates, as well as a phosphotransferase system (PTS). By contrast, most genomes of *Cupriavidus* and *Ralstonia* strains encode putative transporters for carbohydrates.

#### Oxidative stress

Photochemical reactions of UV light with dissolved HS results in the formation of reactive oxygen species (ROS; [27], [28], [29]), which have an inhibitory effect on growth of bacterioplankton [30], [31]. A recent study revealed that at least some *P. necessarius* strains were well adapted to oxidative stress by singlet oxygen [31]. An analysis of the genome of QLW-P1DMWA-1 for traits involved in resistance to oxidative stress revealed no unusual adaptations of the strain. The genome encoded two catalase genes (Pnuc_2044, Pnuc_2053) and two superoxide dismutase genes (Pnuc_1385, Pnuc_1626). In both cases, one of the isoenzymes is putatively exported while the other seems to be a cytoplasmic enzyme. The two encoded superoxide dismutases represent one enzyme of the Fe/Mn and one of the Cu/Zn type. The presence of both two catalase and two superoxide dismutase genes in one genome is not unusual among *Betaproteobacteria*. The analysis of 153 betaproteobacterial genomes revealed the presence of 1 to 6 superoxide dismutase genes per genome. Fifty-five percent of these genomes encoded two such genes, while only 29% encoded only one isoenzyme. Furthermore, the genome encoded a putative glutathione peroxidase (Pnuc_1970), two methionine-S-sulfoxide reductases (Pnuc_0662, Pnuc_0972), two putative rubredoxins (Pnuc_0238, Pnuc_1377), and one putative rubrerythrin (Pnuc_1376).

### Ecological function: major substrate sources

The high average relative and absolute cell numbers of the F10 lineage population in Pond-1 (Fig. 2) indicated that it is rather unlikely that the population is mainly utilizing rare substrate sources. Therefore, the search for their substrate sources should be focused on pools with high substrate concentrations or on pools filled with high fluxes. In case of Pond-1, two basically different substrate pools could potentially provide sufficient amounts of substrates for supporting the large F10 lineage population. On one hand, a low-molecular-weight pool of substances derived from algal or macrophyte (floating mats) primary production and especially algal exudation products could serve as major substrate source of the population [32]. On the other hand, HS forming usually 70–80% of DOC in freshwater systems [33] are present in Pond-1 with estimated average concentrations of about 3–9 mg L^−1^ (average 5 mg L^−1^). We tested experimentally if F10 lineage strains are able to benefit from dissolved HS or algal exudates.

#### Humic substances

Experiments with pure cultures did not indicate a potential for substantial enzymatic degradation and assimilation of HS by strain QLW-P1DMWA-1 (Table S3). Furthermore, screening of the genome for genes potentially involved in HS degradation yielded no indications of such a capability. The genome lacks genes for mono- and dioxygenases that could be involved in cleavage of aromatic rings of HS. This finding is in agreement with the lack of assimilation of phenol and benzoic acid (Table S3). Assimilation of phenylacetate could be explained by cleavage and assimilation of the acetyl residue by an esterase. A gene putatively encoding an exported arylesterase (Pnuc_0938) could be involved in such a reaction. Four other genes were found, which encode putatively exported esterases. However, further assimilation experiments with four other esters (ethylpyruvate, α-terpinylacetate, isoamylacetate, and methylacetate) resulted in significant (t-test, P = 0.01) assimilation only for methylacetate. In order to avoid toxic effects of the tested esters, these experiments were performed with low substrate and NSY medium concentrations (15 mg L^−1^ and 5 mg L^−1^, respectively). Despite the low concentrations, the other three tested substances resulted in significant inhibition effects on the growth of QLW-P1DMWA-1. However, the observed assimilation of phenylacetate and methylacetate may indicate a potential for strain QLW-P1DMWA-1 to utilize at least some aliphatic residues of HS.

Instead of direct enzymatic degradation of HS, the strain could indirectly benefit under *in situ* conditions from HS by utilization of photooxidation products generated by the impact of UV and visible light (VIS) on HS [34], which was indeed suggested for *Polynucleobacter* bacteria previously [15], [17]. We performed two experiments to test this hypothesis. In the first, strain QLW-P1DMWA-1 was grown on light- or dark-treated commercially available HS. In the second, humic water from Pond-1 containing the natural bacterial community (including the F10 lineage) of the pond was incubated under different light treatments (microcosm experiment) and growth of *Polynucleobacter* bacteria and the remaining bacterial community were determined.

In the first experiment increases in cell numbers of QLW-P1DMWA-1 were observed in the light and dark treatments. However, the increase in the dark treatments was only weak and did not significantly differ from the control treatments (data not shown), while the increase in cell numbers in the light treatments significantly differed from the dark and the control treatments (t-test, P<0.005). By considering typical photooxidation-caused production rates of low-molecular-weight (LMW) substances from HS [34], a growth efficiency of 9% – i.e., transformation of 9% of the carbon supplied as HS to cellular carbon – could be calculated for the light-treated HS (Table S3).

**Figure 11:**
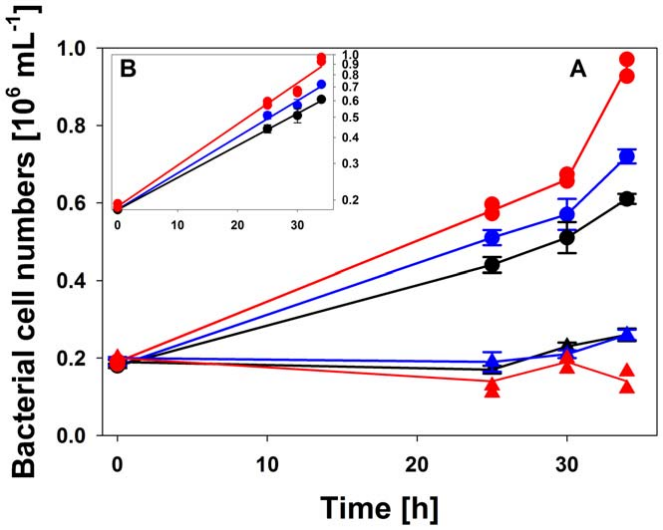
Microcosm experiment on the influence of light on growth of bacteria from Pond-1. Growth of a natural bacterial community originating from Pond-1 was investigated in three different light treatments (UV+VIS, VIS only, and no light). Water from Pond-1 was filtered through membrane filters with a nominal pore size of 0.8 µm (exclusion of potential predators, algae, and other larger organisms) mixed with two volumes of 0.2 µm-filtered pond water (almost free of bacteria) and incubated under *in situ* conditions in floating bottles covered with foil blocking all light (i.e., dark treatment), with foil blocking (>90%) UV light resulting in VIS light only (i.e., VIS only treatment), or in untreated bottles (penetration of >70% UV light), i.e. UV treatment. The bacterial community consisted initially of 48% *P.n.* ssp. *asymbioticus* and 52% other bacteria. The treatments were incubated under *in situ* conditions in the pond for a period of 34 hours. (A) Linear plots of results, (B) Linear regressions of semi-logarithmic plots of the results obtained for *Polynucleobacter*. R^2^ values of the regression lines were 0.999, 0.996, and 0.989 for the dark, VIS, and UV treatments, respectively. Filled circles, *P.n.* ssp *asymbioticus*; filled triangles, other bacteria; red, with UV light; blue, VIS light only, black, no light. All shown data, apart of the data of the UV treatment (red symbols and lines), represent average data and SD (bars) from three replicates. One of three parallels of the UV treatment showed no growth at all. Presentation of average data was therefore omitted and only particular data for each of the two replicates with growth are shown.

In the second experiment (microcosm experiment) the influence of two light qualities (UV+VIS, and VIS only) and the absence of light (dark treatment) on growth of a bacterial community obtained from Pond-1 were investigated. Due to filtration capacity limitations, the absolute and relative *in situ* abundances of F10 lineage bacteria present in the pond at the time the water used for this experiment was sampled could not be determined. However, six days after sampling for the experiment, the relative abundance of *P.n.* ssp. *asymbioticus* cells in Pond-1 was 43.8% of total bacterial numbers and the F10 lineage comprised 21.2% of *P.n.* ssp. *asymbioticus* cells. In the experiment, *P.n.* ssp. *asymbioticus* cell numbers increased exponentially in all three treatments (Fig. 11B), while the other bacteria showed no significant growth (UV treatment) or only very weak and delayed growth (dark and VIS treatments) (Fig. 11A). Interestingly, growth of *Polynucleobacter* bacteria was strongest in the UV treatment (doubling time 15.2 h), but surprisingly, even in the dark treatment *Polynucleobacter* bacteria showed relatively strong growth (doubling time 19.5 h).

#### Algal exudation products

**Figure 12:**
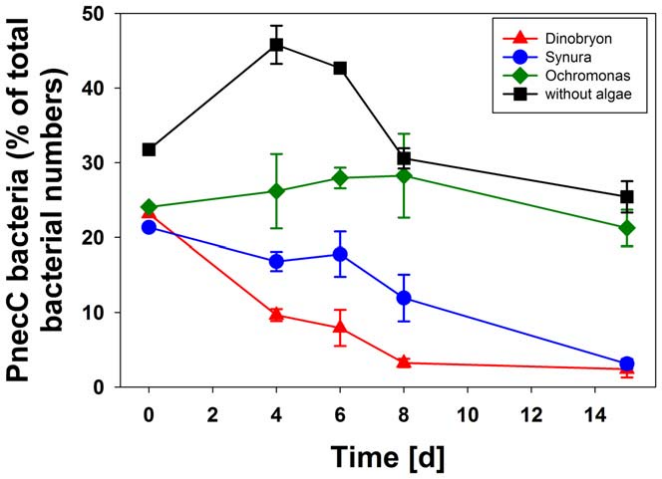
Experiment on potential influence of algal exudates on growth of *P.n.* ssp. *asymbioticus* bacteria. The influence of the presence of growing algae populations on the development of *P.n.* ssp. *asymbioticus* bacteria was investigated in batch culture experiments. Grazer-free (0.8 µm filtered) water from Pond-1 was mixed with different algal cultures representing species typically abundant in Pond-1. The treatments were incubated with continuous illumination for 15 days at a constant temperature of 16°C. The graph shows mean values of relative abundance of *P.n.* ssp. *asymbioticus* bacteria and standard deviations from three replicates. The initial total bacterial numbers in all treatments that had received algae was 4.7×10^6^ cells mL^−1^, and *P.n.* ssp. *asymbioticus* bacteria comprised 23% of bacterial cells. Cell numbers of the three different algal species developed differently over the course of the experiment, but all three showed growth over periods of six to 15 days.

*Polynucleobacter* bacteria sampled from Pond-1 were tested for their growth response to the presence of algae representing abundant phytoplankton species in Pond-1. The results of this experiment are described in detail in Text S3, and Fig. 12 presents a graphical overview. This experiment revealed no indications of measurable growth of F10 lineage strains or other *Polynucleobacter* strains from Pond-1 on algal exudates. In order to further test the utilization of exudation products, experiments on assimilation of glycolate, which is a typical photorespiration exudate of algae [35], [36], were performed with pure cultures of strain QLW-P1DMWA-1. In four experiments, no assimilation of this substance was observed, though slight inhibition of growth was noted. The substrate concentration used (6 mM) was in the range employed by other studies of the utilization of this substance by bacteria, i.e. 1–100 mM [37]. Our own experiments with *Cupriavidus necator* DSM 13513, *C. metallidurans* DSM 2839, and *C. basilensis* DSM 11853 under the same experimental conditions resulted in significant assimilation of glycolate. Furthermore, experiments with 19 other *Polynucleobacter* strains, representing all five currently known species, resulted only in one case in pronounced and, in another case, weak assimilation of this substrate (data not shown). The genome of strain QLW-P1DMWA-1 encoded a putative glycolate oxidase consisting of three subunits (Pnuc_1781, 1782, 1783). The encoded enzyme, however, could also represent a lactate dehydrogenase. In addition, a glycolate permease gene as found in *Ralstonia*/*Cupriavidus* strains is missing in QLW-P1DMWA-1.

## Discussion

Free-living bacteria not associated with any host are traditionally viewed by microbiologists as organisms characterized by high dispersal rates [38], [39] and high potential to adapt to newly invaded environments [40], [41]. Consequently, it could be expected that bacterial populations thriving in particular habitats are characterized by relatively high diversity and frequent changes in population structure, both resulting from frequent invasion by new genotypes and rapid ecological diversification due to adaptation to local ecological conditions. Here we investigated structure and dynamics of a *P.n.* ssp. *asymbioticus* population inhabiting a small acidic pond. Our investigation revealed a population persisting in the habitat over a period of at least seven years and having a relatively stable population structure characterized by a low genetic diversity. On the other hand, this low-diversity population is contributing during the ice-free seasons on average to 11% of the bacterial cells in the water column of the pond. In order to reveal the traits responsible for this ecological success, we characterized the ecology of the population by genome analysis of a representative strain and experimental analysis of the physiology of strains representing the population. These investigations revealed a highly passive lifestyle, which results in exclusion of any opportunity for targeted access and exploitation of trophic hot spots in the water column. The revealed passive lifestyle stands in strong contrast to the ecological success of the population under the volatile environmental conditions of its home habitat, and the low diversity and stable structure of the population contrasts with the expected population dynamics resulting from frequent invasion by new genotypes.

### Genome evolution

Reductive evolution of genome size, also known as genome streamlining, is well documented for ‘true picoplankton’ [42] bacteria inhabiting the upper water layers of the sea [43], [44], [45]. This includes *Candidatus* Pelagibacter ubique [43] affiliated with the ubiquitous SAR11 clade (*Alphaproteobacteria*), as well as some *Cyanobacteria* affiliated with the genus *Prochlorococcus* [44]. These as well as other taxa of the ‘true picoplankton’ fraction of the marine plankton represent abundant groups with cosmopolitan distributions [42] and an oligotrophic physiology [46]. Here we have demonstrated that abundant (Fig. 2) freshwater bacteria passed through a reductive evolutionary process highly similar to that of true marine picoplankton. The ancestors of the investigated *Polynucleobacter* bacteria most likely shared a copiotrophic lifestyle, broad metabolic capabilities, adaptations for dwelling in soils or in association with hosts, as well as a larger genome size with the recent *Burkholderiaceae* taxa (Figs. 5 and 6). Similarly as with *Candidatus* Pelagibacter ubique [43], reductive evolution resulted in a sparser metabolic network with a smaller number of entries for substrates. The reduced catabolic versatility of QLW-P1DMWA-1 is not only indicated by the relatively small proportion of utilized substrates (Fig. 6) and the specialization for carboxylic acids but also in the lower (by about a factor of ten) amount of biomass produced on complex media as compared to other *Burkholderiaceae* species [10]. The high proportions of QLW-P1DMWA-1 genes assigned to EC, Pfam and KEGG categories (Fig. 9) could be interpreted as a trend towards elimination of ‘exotic’ (not well characterized) accessory genes that increase the fitness of the strain only in rare situations. As in true picoplankton bacteria, the decreased catabolic versatility is contrasted with maintenance of basic anabolic pathways, resulting, in the case of QLW-P1DMWA-1, in an almost complete prototrophy. As reported for true picoplankton bacteria [42], [43] motility, chemotaxis, and quorum sensing are absent, and the absolute and relative number of signal transduction genes are low. The genome shares several characteristics with genomes of marine oligotrophs [46]. This includes, for instance, the presence of only a single ribosomal operon, and underrepresentation of genes assigned to the COG categories N (cell motility and secretion), T (signal transduction), V (defense), and K (transcription). An overrepresentation of categories Q (secondary metabolites biosynthesis, transport and catabolism) and I (lipid transport and metabolism), however, was not observed (data not shown). Altogether, streamlining of the QLW-P1DMWA-1 genome resulted in a highly passive oligotrophic lifestyle with little metabolic plasticity.

### Contrast between passive lifestyle and environmental dynamics in the home habitat

A low number of encoded signal transduction genes (Fig. 10) resulting in only minor capabilities to sense changes in environmental conditions and other features of passive lifestyles fit well to true planktonic life in stable environments. *Candidatus* Pelagibacter ubique and *Prochlorococcus* strains inhabit the upper water layers of oceans, where they experience such stable physicochemical conditions [42], [43]. Apart from potential diurnal changes in conditions for photosynthesis and influences of micro-patchiness of nutrient distribution [47], [48], only rather small fluctuations in growth conditions may be experienced by these marine bacteria over time scales of days and weeks. By contrast, the investigated *Polynucleobacter* population persists in a physicochemically and biologically highly dynamic system. Pronounced seasonal differences in oxygen concentration, light availability and temperature occur in annual cycles. Additionally, short-term temporal changes in weather conditions immediately impact the conditions in this small and shallow pond, and sunny weather conditions result in diurnal stratification and mixing cycles (Fig. 3). Mixing of the water column distributes the F10 lineage cells randomly over the depth profile of the pond. The vertical position of particular cells in the water column at the beginning of the stratification process result in strong differences in growth conditions between particular strains for periods of up to 24 hours. Cells placed in the upper water layers experience pronounced increases in water temperature and intensive exposure to sunlight, while cells placed in deeper water layers are much less impacted by solar radiation. As discussed below, such different positions in the water column should result in differences in availability of nutrients (photooxidation products), as well as in different levels of oxidative stress [31]. It is surprising that bacteria characterized by such a passive lifestyle are able to establish under such dynamic environmental conditions such large populations (Fig. 2). Previously published data [6] as well as qPCR data (Jezberová et al., unpublished results) suggest that the F10 lineage is abundant in many habitats with similarly unstable conditions as characterized for Pond-1; the success of this lineage in Pond-1, thus, does not seem to be exceptional. The less extreme genome streamlining of QLW-P1DMWA-1 compared to the three genome-sequenced *Pelagibacter* strains – indicated, for instance by genome sizes of 2.1 Mb versus 1.3–1.5 Mb – may be a consequence of adaptation to a passive lifestyle in a rather unstable environment.

### Ecological function of F10 lineage strains

Results from genome analysis and experimental characterization of metabolic traits indicated that strain QLW-P1DMWA-1 mainly acts as a chemoorganoheterotroph using organic substrates as energy and carbon sources as well as electron donors. In addition, the strain seems able to utilize inorganic sulfur species as electron donors. However, it is not known if reduced sulfur species serve as important energy sources for the F10 lineage population in Pond-1. It is likely that reduced sulfur played no significant role in the microcosm experiment shown in Fig. 11, since the microcosms received only water, and no sediment samples. The experimental conditions thus primarily mimicked the conditions in the water column of the pond but largely excluded the activity of sulfur reducers and fluxes of reduced sulfur from the sediment to the water.

In dystrophic freshwater systems such as Pond-1, the DOC pool of the water body is usually mainly composed of high-molecular-weight (HMW) compounds, i.e. by HS [33]. In contrast to the minor fraction of LMW compounds, the HMW substances represent recalcitrant compounds characterized by long turn-over times [49]. It was found that the absolute and relative abundance of *P.n.* ssp. *asymbioticus* cells in a large set of investigated habitats was positively correlated with concentrations of HS [12]. Explanations for this correlation could be direct or indirect utilization of HS by *P.n.* ssp. *asymbioticus* bacteria. Screening of the genome of QLW-P1DMWA-1 for genes typically involved in microbial degradation of aromatic substances and HS, as well as assimilation experiments with model substances, revealed no indication of any pronounced capabilities in HS degradation (Table S3). Some indications of capabilities of strain QLW-P1DMWA-1 to cleave and utilize at least some aliphatic residues of HS were observed. However, assimilation experiments with HS did not indicate that such capabilities result in significant growth of the strain.

Indirect utilization of HS as photooxidation products was suggested for *Polynucleobacter* bacteria previously [15], [17]. Photooxidation of HS is caused by excitation of HS by UV light and to a lesser extent by visible light [50]. The main products of the photooxidation are LMW organic substances usually mainly composed of carboxylic acids, carbon monoxide, carbon dioxide and other inorganic compounds [34], [51], [52]. Several of the substances assimilated by strain QLW-P1DMWA-1 with high efficiencies in our laboratory experiments, e.g. pyruvate, acetate, and succinate, were reported as photooxidation products [34], and strong uptake of acetate by *P.n.* ssp. *asymbioticus* cells in a humic lake was observed previously [17]. The revealed preference of the strain for carboxylic acids (Fig. 6) fits well in general to the reported spectrum of photooxidation products [34]. However, some substances known to be produced by photooxidation of HS, e.g. citrate, oxalate, or glyoxal, were not assimilated. Experiments with light-treated HS solutions and the microcosm experiment (Fig. 11) further support the notion that F10 lineage bacteria are able to utilize photooxidation products. In the microcosm experiment, the *P.n.* ssp. *asymbioticus* population responded best to the UV treatment while the other bacteria showed the opposite response. Surprisingly, *P.n.* ssp. *asymbioticus* bacteria also grew well but more slowly in the dark treatment. This could indicate that despite the storage of the water samples used for the experiment in the dark for 24 h, the water had still contained enough substrates available for *P.n.* ssp. *asymbioticus* and also other bacteria. The delayed growth of the other bacteria could have been caused either by the presence of reactive oxygen species (ROS) with long half-lives, e.g. hydrogen peroxide (H_2_O_2_), for which half-lives in lake water of 10–22 h have been determined [53], or longer-lasting inhibitory effects caused by singlet oxygen (^1^O_2_) through direct cell damage by oxidation of lipids, nucleic acids and proteins [54]. Both variants of ROS are formed during photooxidation of HS [27], [28], [29]. Thus, the delayed growth of the other bacteria could have been a result of the environmental conditions at the time the water samples used in the experiment were taken.

Even algae in fairly good physiological state release a part of their photosynthetic products to the surrounding water [35] under natural conditions. These released organic substances represent an important carbon and energy source for freshwater and marine bacterioplankton [55]. The quality and quantity of the released organic material is species-specific and strongly influenced by growth conditions and growth phase of the algae. However, carbohydrates (mainly polysaccharides) and glycolate usually constitute the major fraction of the released substances [35]. Sugar alcohols, other organic acids, peptides and amino acids were found to be released by many algal species but constituted frequently only small fractions of algal exudates. The most frequently released amino acids were found to be Ser, Gly, Lys, Ala, Glu, Asp, Orn, and His. Our assimilation experiments indicated that strain QLW-P1DMWA-1 did not – or only to a low extent – assimilate most of the mentioned algal exudation products. Several monosaccharides and glycolate were not utilized, whereas some sugars were utilized with low efficiency. The almost complete lack of genes putatively encoding transporters for carbohydrates, as well as the lack of genes for specific phosphorylation of monosaccharides suggests that strain QLW-P1DMWA-1 is not adapted for utilization of glucose, fructose, mannose and other carbohydrates. The observed weak utilization of such substances could represent laboratory artifacts caused by high substrate concentrations in the assimilation experiments. Interestingly, Paver & Kent [36] suggested that *Polynucleobacter* bacteria represent a large part of the freshwater bacterioplankton with the genetic potential to use glycolate. They used bacteria-specific primers for specific amplification of glycolate oxidase genes (glcD) encoding one subunit of the glycolate oxidase, which is a key enzyme in the assimilation of glycolate [37]. Forty-eight percent of the glcD sequences obtained from five lakes clustered with the putative glcD gene (Pnuc_1781) of strain QLW-P1DMWA-1 [36].

Altogether, our results do not indicate that *P.n.* ssp. *asymbioticus* strains affiliated with the F10 lineage are specially adapted to utilization of algal exudation products. Neither the experimentally revealed assimilation patterns nor the growth experiment performed in the presence of algae (Fig. 12) indicate such an adaptation. In addition, besides the presence of putative glycolate oxidase genes, there is a lack of genomic data further supporting this kind of adaptation. However, we cannot rule out that other lineages affiliated with *P.n.* ssp. *asymbioticus* or other *Polynucleobacter* taxa mainly grow on substances released by phytoplankton. Previous investigations on *P. acidiphobus*/*P. difficilis* assemblages (PnecB) in Lake Mondsee indicated a relationship to phytoplankton dynamics [23], and recent experimental investigations support this hypothesis [3]. In contrast to our investigations on the F10 lineage, other studies on different freshwater bacteria, i.e. strains affiliated with the important genus *Limnohabitans*, resulted in a more obvious match between substrate assimilation pattern [56], successful growth on algal exudates [3], and correlation between phytoplankton and *Limnohabitans* dynamics [57], [58]. Even weak glycolate assimilation was demonstrated for *Limnohabitans planktonicus* [56].

**Figure 13:**
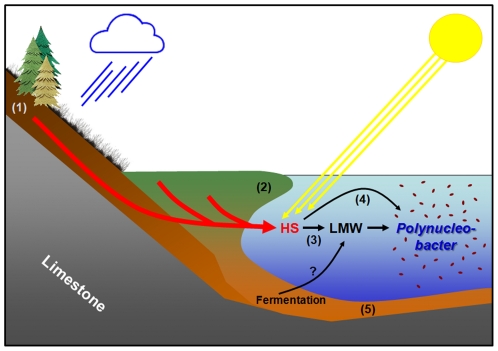
Conceptual model on carbon fluxes utilized by the F10 lineage population in Pond-1. The model suggests origin and transformation of major carbon sources utilized by the F10 lineage population inhabiting Pond-1. Humic substances (HS) leached from soils (1) and floating mats (2) (*Schwingrasen*, formed mainly of *Sphagnum* and *Carex*) are transported by percolating water to the pond. *P.n.* ssp. *asymbioticus* bacteria affiliated with the F10 lineage mainly utilize low-molecular-weight (LMW) photooxidation products of HS (3). Direct utilization of aliphatic residues of HS by enzymatic cleavage is also expected to take place (4). Another potential substrate source could be fermentation products released by bioturbation and other processes from the sediment (5) to the water column of the pond.

Based on the presented results, we propose a model of the ecological function of the F10 lineage population in Pond-1 that is centered on the utilization of photooxidation products of HS (Fig. 13). The hydrological conditions of Pond-1 indicated that the majority of HS dissolved in the water body is of terrestrial origin. HS are transported to the pond by water percolating from the surrounding slopes, as well as from the *Sphagnum*- and *Carex*-dominated floating mats. In the pond, HS are temporarily exposed to solar radiation and a slow photodegradation takes place. The resulting LMW substances are utilized by the F10 and potentially also by the few other *P.n.* ssp. *asymbioticus* lineages present in the pond [4]. Due to the limited versatility caused by the evolutionary genome streamlining, the F10 lineage bacteria can most likely consume only a fraction of the released photooxidation products. This limited versatility may enable the coexistence of different lineages by substrate partitioning [59]. For instance, the F10 lineage bacteria seem unable to consume citrate, which was reported to be a product of photodegradation [34]. In addition to utilization of photooxidation products, F10 lineage bacteria may cleave and utilize aliphatic residues of HS. The LMW pool exploited by the F10 lineage bacteria may also be filled by other fluxes, e.g. the release of fermentation products from anoxic sediment or the cleavage of HS by microbial exoenzymes released by other organisms. Additionally, even algal exudation may deliver some substances utilized by this lineage. All data obtained so far indicate that the F10 lineage bacteria float passively in the water column of the pond and mainly wait for the delivery of substrates by the photooxidation processes. The performed predation experiments suggest that the *P.n.* ssp. *asymbioticus* population including the F10 lineage serves as a food source for heterotrophic and mixotrophic protists, which should results in a transfer of HS-derived organic material in the pelagic food web of the pond.

### Why is the passive lifestyle of the investigated population so successful?

The above-presented hypothesis on the ecological function of the investigated F10 lineage population gives no indication at all that these bacteria represent specialists able to monopolize a trophic niche. In contrast, it has to be assumed that a broad diversity of other bacteria should also be able to exploit the substrate sources utilized by the F10 lineage population. Most likely, non-trophic adaptations - or at least combinations of trophic and non-trophic adaptations - are responsible for the ecological success of the population. What kind of adaptations could make the F10 lineage population so successful in Pond-1 and other *P.n.* ssp. *asymbioticus* populations so successful in other habitats? Results from several studies indicate that a rather passive and oligotrophic lifestyle is a common trait of most or all *P.n.* ssp. *asymbioticus* bacteria (e.g. [10], [11], [15]). Furthermore, the commonly relatively low G+C content of *Polynucleobacter* genomes (<50 mol% vs. >60 mol% for the majority of other *Burkholderiaceae*) as well as previous estimations of genome sizes [7] suggest that reduced genome sizes represent a common feature of *Polynucleobacter* bacteria. Interestingly, the sibling group of *Polynucleobacter* formed by the two genera *Cupriavidus* and *Ralstonia* (Fig. 8) is absent or plays only a minor role in the water columns of freshwater bodies [1], [2]. Members of these two genera are generally characterized by genome sizes about three times the size of *Polynucleobacter* genomes. If microevolutionary adaptations, e.g. adaptations to the spectrum of major substrates available in the water columns of freshwater systems, were enough to become successful planktonic players, one would expect *Cupriavidus*/*Ralstonia*-like bacteria to contribute significantly to the composition of freshwater bacterioplankton. Instead, the genome-streamlined sibling group *Polynucleobacter* is the one that represents a prominent fraction of freshwater bacterioplankton [12]. Macroevolutionary genome economization seems to be a prerequisite for a successful colonization of the water column of freshwater systems by *Burkholderiaceae* bacteria. It is believed that, in prokaryotes, small genome size is a prerequisite for maintenance of small cell sizes by metabolically active and growing cells [42]. Small cell sizes are known to be advantageous in substrate acquisition due to the increased surface-to-volume ratio [60], as well as in the interaction with many protistan predators [61], [62] and viruses [63]. Future investigations will have to reveal if optimization of substrate uptake or reduction of predation mortality is the more important driver in the evolution of *P.n.* ssp. *asymbioticus* bacteria. However, it is likely that additional but so far undiscovered or only superficially investigated adaptations also contribute to the ecological success of these bacteria. Resistance to oxidative stress [31] could be one such trait but does not have to be the only relevant adaptation. The passive lifestyle of the F10 lineage bacteria could, rather, be interpreted as a byproduct of the genome size reduction than as a strategy responsible for the success of the population.

### Could the obtained genomic and ecological insights be generalized to other *P. necessarius* lineages?

Currently, we can only speculate if generalization of the obtained insights in the ecology and adaptation of the investigated lineage to other lineages of *P.n.* ssp. *asymbioticus* or other *Polynucleobacter* species is suitable. To what extent differences in adaptations are involved in the ecological diversification of *P.n.* ssp. *asymbioticus* bacteria is still unknown [6]. This diversification may be restricted to non-trophic adaptations such as pH or temperature adaptation. In this case, it could be that all *P.n.* ssp. *asymbioticus* strains exploit more or less the same trophic niche, i.e. utilization of LMW products of HS photodegradation. On the other hand, adaptation to different trophic niches can currently not be excluded. Reports on photoheterotrophy in *Polynucleobacter* bacteria [19] and indications of potential utilization of major algal exudation products [36] may suggest a pronounced trophic diversification.

### Conclusions

Evolutionary genome streamlining of F10 lineage bacteria resulted in a highly passive lifestyle so far known only among free-living bacteria of pelagic marine taxa (true picoplankton bacteria) dwelling in environmentally stable, nutrient-poor, off-shore systems. Surprisingly, such a lifestyle is also successful in a highly dynamic and nutrient-richer environment such as the water column of the investigated pond. Obviously, neither metabolic and ecological versatility nor a pronounced microdiversity of populations are prerequisites for long-lasting establishment of abundant bacterial populations under highly dynamic environmental conditions. We are only just beginning to understand the diversity and ecology of *Polynucleobacter* bacteria, therefore, caution should be exercised when generalizing the obtained insights in ecology and adaptation of the investigated lineage to other *Polynucleobacter* lineages.

## Materials and Methods

### Sampling of Pond-1

The pond was sampled over a period of six years (autumn 2003 to autumn 2009), as described elsewhere [4]. Most of the samplings were performed during the vegetation periods (May to November), but two samplings were performed in deep winter (2004 and 2008). No specific permits were required for the described field studies.

### Quantification of bacteria

Total bacterial numbers were determined by epifluorescence microscopic analysis of DAPI-stained samples as described previously [4]. Total numbers of *P. necessarius* bacteria were determined by fluorescence *in situ* hybridization (FISH) using the specific probe PnecC-16S-445 as described previously [4]. The relative contribution of the *Polynucleobacter* F10 lineage to the total number of *P. necessarius* bacteria was determined by quantitative PCR (qPCR). Quantitative PCR was performed on an Eppendorf Mastercycler (ep realplex Thermal Cyclers) using TaqMan probes labeled with 6-FAM (5′-end) and 5-TAMRA (3′-end) and template DNA extracted from water samples as described previously [6]. We have designed two quantitative PCR assays, one specific for the F10 lineage, and one for the entire taxon *P. necessarius*. The F10 lineage assay detected a 97 bp fragment and was performed with the forward primer G1_TF (5′-GCTGTTTAGGTGAAGAAAGTGTGACA-3′), reverse primer G1_TR (5′-CTTTCTGTAATGGCGCTTTCTG-3′) and probe G1_TM (5′-6FAM-TTTGCCGAACACCCGCTTATCAATACACTG-TMR-3′). The *P. necessarius* assay detected a fragment of a total length of 142 bp and consisted of the forward primer C_TF (5′-GRGTAAAGATTGAATCATCAATCAG-3′), reverse primer C_TR (5′-CTATAACGAGCACCATTGCTAGY-3′) and the probe C_TM (5′-6FAM-CTTACAGTTTGGATTACGGCAAACATGTC-TMR-3′). The 12 µL PCR reactions contained PCR water, 1× buffer (TaqMan Universal PCR Master Mix - Applied Biosystems), 900 nM forward primer, 900 nM reverse primer and 150 nM probe. The PCR cycle consisted of 10 min at 95°C followed by 50 cycles: 95°C for 15 s, 60°C for 60 s. Fluorescence signal results were analyzed using the Realplex software. CT value threshold was set for 100, and CT values were recalculated to gene copies using standards. The ratio P_F10_/P_all_ of copy numbers of markers specific for the F10 lineage (P_F10_) and markers specific for all *P. necessarius* (P_all_) was determined. Absolute numbers of F10 lineage cells were calculated by multiplication of the absolute number of *P. necessarius* cells, as determined by FISH, by the ratio P_F10_/P_all_.

### Grazing experiments

Two grazing experiments with two different kinds of fluorescently labeled bacteria (FLB) were performed for estimation of grazing rates of protists on the entire bacterial community and the *Polynucleobacter* assemblage in Pond-1. FLB were produced using a sample of natural bacterioplankton (Římov Reservoir, Czech Republic), as well as a pure culture of strain QLW-P1DMWA-1 by following previously published protocols ([64]; for specific details see [65]). In each experiment FLB (15–20% of total bacterial numbers in a sample) were added to a water sample from Pond-1 (20 September 2005) and incubated under *in situ* conditions (temperature 9°C) in the laboratory. Subsamples were taken at 10–15 minute intervals (ciliates) and 60 minute intervals (flagellates) after addition of the FLB and fixed by adding 0.5% of alkaline Lugol's solution, immediately followed by 2% (vol/vol) (final concentration) formaldehyde and several drops of 3% (wt/vol) sodium thiosulfate to clear the color of the Lugol's solution [64]. The numbers of ingested FLB per protistan cell were enumerated using an epifluorescence microscope [65]. Food vacuole content of the protozoa was also checked for presence of FISH-positive cells using the FISH probe PnecC-16S-445 [4] as described elsewhere [66].

### Isolation of *Polynucleobacter* strains

Bacterial strains were either isolated by the filtration acclimatization method [67] or by the dilution acclimatization method [4]. Pure cultures of strains were stored at −70°C. *Polynucleobacter* strains and F10 lineage strains were identified by sequencing of the 16S–23S ITS [4], [6].

### Multilocus sequence analyses of F10 strains

The complete genome sequence (accession number CP000655, [22]) of *P.n.* ssp. *asymbioticus* strain QLW-P1DMWA-1 [4], [10] was used to develop primers for multilocus sequence analyses (Table S4) of F10 strains isolated from Pond-1. Furthermore, all twelve F10 strains (including QLW-P1DMWA-1) isolated from the pond were characterized by genetic fingerprinting with three independent methods, as described previously [4].

### Phylogenetic analysis

The phylogenetic position of the genome-sequenced strain QLW-P1DMWA-1 was reconstructed using a multilocus sequence analysis approach. The sequences of eight protein-encoding housekeeping genes (Table S5) of the strain and ten genome-sequenced reference strains (Table S6) were used for this analysis. Phylogenetic trees were constructed by the neighbor-joining (NJ), the maximum likelihood (ML), and the maximum parsimony (MP) methods, using the software MEGA4 and MEGA5 [68]. Tree constructions were based on either nucleotide sequences or amino acid sequences. For comparison, the phylogeny of the 16S rRNA gene of the eleven strains was reconstructed using the same treeing methods.

### Comparative analysis of genomic traits

Comparative analyses of the genome of strain QLW-P1DMWA-1 were mainly performed using the integrated microbial genomes (IMG) system [24]. Data on signal transduction genes of the strain and reference bacteria were obtained from the Microbial Signal Transduction Database MIST2 [69]. A functional interpretation of putative transporter proteins was performed by comparing the amino acid sequences of putative transporter genes to the database TransportDB [70]. Because this comparative analysis, for the majority of putative transporters, gave no indications of their specific putative function (i.e., of their specific putative substrates), putative transporter genes were assigned to seven more general categories of putative functions (i.e., import of amino acids, import of carbohydrates, import of carboxylic acids, import of other organic substances, import of inorganic nutrients, export and efflux, and unknown transport function).

### Substrate assimilation tests with strain QLW-P1DMWA-1

Assimilation tests with a total of 93 substrates were performed in batch culture experiments. Previous experiments have revealed that growth of QLW-P1DMWA-1 on single carbon and energy sources is unreliable and weak [10]. Therefore, assimilation experiments were performed by comparing optical density (OD_575 nm_) values established in one-tenth-strength (0.3 g L^−1^) liquid NSY medium [67] with and without 0.5 g test substance L^−1^ (pH 7.2). The NSY medium was supplemented with thiamine (80 µg L^−1^), biotine (0.4 µg L^−1^) and cyanocobalamine (0.4 µg L^−1^). Photometrically determined ΔOD_575 nm_ values (i.e., OD_NSY+substrate_ – OD_NSY_) represented biomass generated by the strain by utilizing the tested substrate. Growth efficiencies – i.e. the fraction (%) of test substrate carbon transformed to bacterial biomass carbon – for the various tested substrates were calculated by transferring ΔOD_575 nm_ values to cell numbers based on an empirical conversion factor, multiplication of cell numbers with epifluorescent microscopically determined average cell volumes, and conversion of cell volumes to carbon units using the conversion factor 308 fg C µm^−3^ suggested by Fry [71]. According to Loferer-Krößbacher et al. [72], this factor is the most probable volume-to-carbon conversion factor for cells with sizes in the range observed for strain QLW-P1DMWA-1. Assimilation experiments were performed in triplicates at room temperature, and for each experiment OD values or cell numbers (see below) were determined twice after 7 and 11 days of incubation. Cultures were carefully checked for contaminations by streaking samples taken at the start and end of the experiments on agar plates. Experiments lacking positive ΔOD_575 nm_ values were repeated at least once. Experiments resulting in negative ΔOD_575 nm_ values, which potentially indicates toxic effects of the test substance on the bacterial strain, were repeated with decreased concentrations of test substances (15 mg L^−1^) and NSY medium (5 mg L^−1^). In these experiments, growth was recorded by epifluorescence microscopical cell counts. Assimilation of four esters was exclusively tested at these low substrate concentrations. The tested substances are listed in Table S3.

### Experiment on influence of light conditions on growth of strain QLW-P1DMWA-1 on dissolved humic acids

For testing the utilization of photooxidation products of humic substances (HS) by strain QLW-P1DMWA-1, commercially available HS (Sigma-Aldrich) were used. Light treatment and growth experiment were performed separately. HS were dissolved (500 mg L^−1^) in Milli-Q water, filled in 1 L Pyrex bottles (for light transmission properties of the bottle see below), and the solutions were sterilized by microwaving and exposed for three days to natural sunlight (February 2008, sunny weather; total light exposure was 21 hours). Triplicated dark (covered with aluminum foil) and light treatments (no cover) were exposed in parallel. Samples for measurement of fluorescence excitation emission matrix (EEM) were taken before and after exposure. For testing growth of QLW-P1DMWA-1, nine bottles were set up and inoculated with the strain. Three bottles received aliquots from the light-treated HS solutions, three received aliquots from the dark-treated bottles, and three received no HS (negative control). All bottles contained IBM medium (not supporting growth of the bacteria) supplemented with a vitamin mix (see above), and the calculated HS concentrations were 30 mg L^−1^. Bottles were incubated in the dark at room temperature for 18 days. Bacterial cell numbers and cell sizes were determined microscopically.

### Experiment on influence of light conditions on growth of the natural *Polynucleobacter* population of Pond-1

Water sampled from Pond-1 before sunrise was filtered either through 0.8 (removal of algae, protistan predators and other larger particles) or 0.2 µm (removal of almost all bacteria and larger particles) polycarbonate membrane filters and stored in the dark for 24 h to minimize inhibitory effects of potentially present reactive oxygen species (ROS), e.g. H_2_O_2_ [30]. The next day, the differently filtered water samples were mixed in a ratio of 1∶2 (0.8 µm∶0.2 µm filtrates), and 150 mL volumes of the mixture were distributed in 1 L borosilicate bottles. Three different light treatments were created by covering bottles with aluminum foil (no light), by covering bottles with UV filter foil (e-color #226, Rosco) (visible light only), and by using uncovered bottles (UV and visible light). Transmission measurements with a Xenon (75 W) light source revealed that the UV filter foil absorbed >90% of light in the 350–380 nm range (UV) and 10% in the 390–780 nm range (visible light, VIS). The uncovered borosilicate bottles absorbed 65–70% of UV light and 65% (380 nm) to 10% (700 nm) of the visible light. The transmissibility of the bottles increased steadily from 380 to 700 nm. Three bottles of each treatment were incubated in the pond for 34 h. During the incubation, the bottles floated at the water surface and were kept in position by a rope.

### Experiment on potential utilization of algal exudates as major substrate source

Batch experiments with a natural bacterial community (including the F10 lineage) obtained from Pond-1 and three species of algae were performed under controlled laboratory conditions. Non-axenic cultures of three algal species, i.e. non-phagotrophic *Synura* sp. LO2 34 K-F, putatively mixotrophic *Dinobryon* sp. LO2 26 K-S, and putatively mixotrophic *Ochromonas* sp. LO2 44 K-D, isolated by Steffen Jost from a dystrophic pond located at a distance of 120 m from Pond-1 were used as potential substrate producers for bacteria. The two ponds shared highly similar physicochemical characteristics (Hahn et al, unpublished data), and all three taxa represented typical species of both ponds (Alexandra Pitt, personal communication). Bacteria from Pond-1 (sample of 3^rd^ November 2007) were separated from protistan predators and other eukaryotes by filtration through a 0.8 µm membrane filter [73]. Four treatments, of three different algal species and one control without algae, were each set up in triplicates. In the treatments with algae, 200 ml of 0.8 µm filtered pond water was mixed with 30 ml of algal cultures and distributed equally in three 100 mL Erlenmayer flasks. The controls received no algae. The treatments were incubated in a cultivation room at 16°C with continuous illumination. Samples for determination of total bacterial numbers, *Polynucleobacter necessarius* numbers, and algal numbers were taken after 0, 4, 6, 8 and 15 days of incubation. Algal cells were counted after fixation with Lugol's solution using an inverted light microscope [74].

### DNA sequences accession numbers

The GenBank/EMBL/DDBJ accession numbers for the sequences used in the microdiversity study on the F10 lineage population in Pond-1 are HE646440 - HE646569, AJ879799, and AJ879800. All relevant sequences of strain QLW-P1DMWA-1 were taken from the deposited genome sequence (for gene tags see Table S4).

## Supporting Information

Table S1 Current taxonomy of *Polynucleobacter* bacteria. The genus *Polynucleobacter* currently harbors five species (Hahn et al., 2009, 2010, 2011a, 2011b, 2012). These five species represent the four *Polynucleobacter* tribes (PnecA to PnecD) suggested by the taxonomy in Newton et al. (2011). Some of the strains affiliated with the subspecies *P. necessarius asymbioticus* were recently assigned to lineages putatively representing differently adapted ecotypes (Jezbera et al., 2011). Lineages F10, F5 and F4 inhabit Pond-1 while other lineages and genotypes are absent from this habitat (Hahn et al., 2005). The populations of these three lineages dwelling in Pond-1 were previously described as G1, G2 and G3 populations (Hahn et al., 2005). The current study is focusing exclusively on the F10 lineage (G1) population of Pond-1. The subspecies *P. necessarius* ssp. *necessarius* contains exclusively endosymbionts of ciliates (Hahn et al., 2009), thus contains only strains fundamentally differing in lifestyle from all other currently established *Polynucleobacter* taxa (Vannini et al., 2007).
(DOCX)

Table S2 Abbreviations
(DOCX)

Table S3 Results of the assimilation experiments performed with 93 substrates. Substrates that did not support growth of the strain are highlighted in red. Substrates yielding growth efficiencies ≥5% are highlighted in green. Growth efficiencies were not determined for five of the listed substrates.
(DOCX)

Table S4 Loci used for the multilocus sequence analysis of the eleven F10 lineage strains isolated from Pond-1 (compare Fig. 5). The sequences of Pnuc_1240 were not considered for the calculation of the tree shown in Fig. 5.
(DOCX)

Table S5 Loci used for the multilocus sequence analysis shown in Fig. 8.
(DOCX)

Table S6 Genomes and strains considered for the multilocus sequence analysis presented in Fig. 8.
(DOCX)

Table S7 Bootstrap values obtained in phylogenetic reconstructions using the concatenated amino acid (AA) or nucleotide sequences (nt) of eight housekeeping genes (Fig. 8). For comparison, phylogenetic reconstructions solely based on the 16S rRNA gene sequences of the same set of organisms were performed (tree not shown). Note the strong support for node E placing the *Polynucleobacter* strain next to the *Ralstonia*/*Cupriavidus* clade.
(DOCX)

Text S1 Lack of genes well known from other *Burkholderiaceae* bacteria.
(DOCX)

Text S2 Search for genes involved in adaptations to a planktonic lifestyle.
(DOCX)

Text S3 Experiment on growth support of *Polynucleobacter* bacteria by algal exudates.
(DOCX)
