# Supplementary material for: The Passive Yet Successful Way of Planktonic Life: Genomic and Experimental Analysis of the Ecology of a Free-Living Polynucleobacter Population
Source: PLoS One. 2012 Mar 20;7(3):e32772. doi: 10.1371/journal.pone.0032772 (PMC3308952; doi:10.1371/journal.pone.0032772)
Supplement: Table S1 — Current taxonomy of Polynucleobacter bacteria. The genus Polynucleobacter currently harbors five species (Hahn et al., 2009, 2010, 2011a, 2011b, 2012). These five species represent the four Polynucleobacter tribes (PnecA to PnecD) suggested by the taxonomy in Newton et al. (2011). Some of the strains affiliated with the subspecies P. necessarius asymbioticus were recently assigned to lineages putatively representing differently adapted ecotypes (Jezbera et al., 2011). Lineages F10, F5 and F4 inhabit Pond-1 while other lineages and genotypes are absent from this habitat (Hahn et al., 2005). The populations of these three lineages dwelling in Pond-1 were previously described as G1, G2 and G3 populations (Hahn et al., 2005). The current study is focusing exclusively on the F10 lineage (G1) population of Pond-1. The subspecies P. necessarius ssp. necessarius contains exclusively endosymbionts of ciliates (Hahn et al., 2009), thus contains only strains fundamentally differing in lifestyle from all other currently established Polynucleobacter taxa (Vannini et al., 2007). (DOCX) [file pone.0032772.s001.docx]

}

*Polynucleobacter acidiphobus* (PnecB1)

(PnecB)

*Polynucleobacter difficilis* (PnecB2)

*Polynucleobacter cosmopolitanus*  (PnecD)

***Polynucleobacter necessarius***  (PnecC)

***Polynucleobacter necessarius* subsp. *asymbioticus***

**Lineage F10 (including G1)**

Lineage F5 (including G2)

Lineage F4 (including G3)

Other lineages

Strains not yet assigned to any lineage

*Polynucleobacter necessarius* subsp. *necessarius*

*Polynucleobacter rarus* (PnecA)

**References**

**Hahn MW, Lang E, Brandt U, Lünsdorf H., Wu QL, et al. (2010)** *Polynucleobacter cosmopolitanus* sp. nov., free-living planktonic bacteria inhabiting freshwater lakes and rivers. Int J Syst Evol Microbiol **60:** 166-173.

**Hahn MW, Lang E, Brandt U, Spröer C (2011b)** *Polynucleobacter acidiphobus* sp. nov., a representative of an abundant group of planktonic freshwater bacteria. Int J Syst Evol Microbiol **61:** 788-794.

**Hahn MW, Lang E, Brandt U, Wu QL, Scheuerl T (2009)** Emended description of the genus *Polynucleobacter* and the species *P. necessarius* and proposal of two subspecies, *P. necessarius* subspecies *necessarius* subsp. nov. and *P. necessarius* subsp. *asymbioticus* subsp. nov. Int J Syst Evol Microbiol **59:** 2002-2009.

**Hahn MW, Lang E, Tarao M, Brandt, U (2011a)** *Polynucleobacter rarus* sp. nov., a free-living planktonic bacterium isolated from an acidic lake. Int J Syst Evol Microbiol **61:** 781-787.

**Hahn MW, Minasyan A, Lang E, Koll U, Spröer C (2012)** *Polynucleobacter difficilis* sp. nov., a planktonic freshwater bacterium affiliated with subcluster B1 of the genus *Polynucleobacter*. Int J Syst Evol Microbiol. **62:** 376–383.

**Hahn MW, Pöckl M, Wu QL (2005)** Low intraspecific diversity in a *Polynucleobacter* subcluster population numerically dominating bacterioplankton of a freshwater pond. Appl Environ Microbiol **71:** 4539–4547.

**Jezbera J, Jezberová J, Brandt U, Hahn MW (2011)** Ubiquity of *Polynucleobacter* *necessarius* subspecies *asymbioticus* results from ecological diversification. Environ Microbiol **13:** 922–931.

**Newton, RJ, Jones SE, Eiler A, McMahon KD, Bertilsson S (2011)** A guide to the natural history of freshwater lake bacteria. Microbiol Mol Biol Rev **75:** 14-49.

**Vannini C, Pöckl M, Petroni G, Wu QL, Lang E, et al. (2007)** Endosymbiosis in statu nascendi: Close phylogenetic relationship between obligately endosymbiotic and obligately free-living *Polynucleobacter* strains (*Betaproteobacteria*). Environ Microbiol **9:** 347-359.
