# Supplementary material for: The Passive Yet Successful Way of Planktonic Life: Genomic and Experimental Analysis of the Ecology of a Free-Living Polynucleobacter Population
Source: PLoS One. 2012 Mar 20;7(3):e32772. doi: 10.1371/journal.pone.0032772 (PMC3308952; doi:10.1371/journal.pone.0032772)
Supplement: Table S2 — Abbreviations (DOCX) [file pone.0032772.s002.docx]

**ABC transporters**, ATP-binding cassette transporter, family of transporter proteins

**aka**, also known as

**Ala**, alanine

**Asp**, aspartate

**bp**, base pairs

**COG**, clusters of orthologous groups of proteins

**CRISPR**, clustered regularly interspaced short palindromic repeats

**CT value**, cycle threshold value

**DAPI**, 4',6-diamidino-2-phenylindole, a fluorescent dye

**DNA**, deoxyribonucleic acid

**DOC**, dissolved organic carbon

**DSM**, Deutsche Sammlung von Mikroorganismen (German Collection of Microorganisms and Cell Cultures, DSMZ)

**EC**, Enzyme Commission

**EEM**, fluorescence excitation emission matrix

**F10 lineage**, phylogenetic group within PnecC (see Supplementary Materials Table S1)

**FAM**, **6FAM**, 6-carboxyfluorescein, a fluorescent dye

**FISH**, fluorescence *in situ* hybridization

**FLB**, fluorescently labeled bacteria

**G+C content** (= GC value), guanine-cytosine content of DNA (usually given as mol%)

**G1**, genotype 1, subgroup of F10 lineage (see Supplementary Materials Table S1)

**GE**, growth efficiency

**glcD**, glycolate oxidase gene

**glnA**, glutamine synthetase gene

**Glu**, glutamate

**Gly**, glycine

**His**, histidine

**HMW**, high-molecular-weight

**HNF**, heterotrophic nanoflagellates

**HS**, humic substances

**IBM**, inorganic basal medium

**IMG system**, Integrated Microbial Genomes system

**ITS**, intergenic transcribed spacer (aka, internal transcribed spacer)

**kb**, kilo base pairs (10^3^ bp)

**KEGG**, Kyoto Encyclopedia of Genes and Genomes

**LMW**, low-molecular-weight

**Lys**, lysine

**Mb**, mega base pairs (10^6^ bp)

**ML**, maximum likelihood

**MP**, maximum parsimony

**NJ**, neighbor-joining

**NSY medium**, nutrient broth-soytone-yeast extract medium

**OD**, optical density

**ORF**, open reading frame

**Orn**, ornithine

**PCR**, polymerase chain reaction

**Pfam**, database of protein families

**PnecA**, **PnecB**, **PnecC**, **PnecD**, phylogenetic clusters representing subgroups within the genus *Polynucleobacter* (see Supplementary Materials Table S1)

**PnecC**, *Polynucleobacter* cluster C, currently more or less identical with the species *P. necessarius*

**PTS**, phosphotransferase system

**QLW-P1DMWA-1**, strain designation of the genome sequenced bacterium

**qPCR**, quantitative PCR

**RLBH**, reverse line blot hybridization

**ROS**, reactive oxygen species

**rRNA**, ribosomal ribonucleic acid

**SAR11**, clade of marine *Alphaproteobacteria* including *Candidatus* Pelagibacter ubique

**Ser**, serine

**SOX genes**, genes of the SOX locus encoding parts of the sulfur-oxidizing (Sox) enzyme system first characterized in *Paracoccus*

**ssp**., subspecies

**TAMRA**, **5-TAMRA**, 5-carboxytetramethylrhodamine, a quencher

**TCA,** tricarboxylic acid cycle

**tRNA,** transfer ribonucleic acid

**UV**, ultra violet light

**VIS**, visible light
