# Supplementary material for: The Passive Yet Successful Way of Planktonic Life: Genomic and Experimental Analysis of the Ecology of a Free-Living Polynucleobacter Population
Source: PLoS One. 2012 Mar 20;7(3):e32772. doi: 10.1371/journal.pone.0032772 (PMC3308952; doi:10.1371/journal.pone.0032772)
Supplement: Table S3 — Results of the assimilation experiments performed with 93 substrates. Substrates that did not support growth of the strain are highlighted in red. Substrates yielding growth efficiencies ≥5% are highlighted in green. Growth efficiencies were not determined for five of the listed substrates. (DOCX) [file pone.0032772.s003.docx]

|  | **Substrate** | **Growth efficiency** |
| --- | --- | --- |
|  |  | **(%)^#^** |
|  |  |  |
| **Carbohydrates and derivatives** | | |
|  | D-Glucose | **0.4** |
|  | D-Arabinose | **0.0** |
|  | D-Erythrose | **0.0** |
|  | D-Lyxose | **1.0** |
|  | D-Galactose | **0.6** |
|  | D-Mannose | **1.2** |
|  | D-Xylose | **0.0** |
|  | D-Fructose | **0.6** |
|  | D-Ribose | **0.0** |
|  | L-Fucose | **0.5** |
|  | L-Sorbose | **0.0** |
|  | L-Rhamnose | **1.7** |
|  | N-Acetyl-D-glucosamine | **0.4** |
|  | Pectin | + **^&^** |
|  |  |  |
| **Alcohols, sugar alcohols, sugar acids** | | |
|  | D-Galacturonic acid | **1.4** |
|  | D-Gluconate | **0.0** |
|  | D-Sorbitol | **0.4** |
|  | Ethanol | **0.0** |
|  | Glycerate | **0.0** |
|  | Glycerol | **0.0** |
|  | Methanol | **0.0** |
|  |  |  |
| **Amino acids** | |  |
|  | Glycine | **0.0** |
|  | L-Alanin | **0.0** |
|  | L-Arginie | **0.0** |
|  | L-Asparagine | **0.5** |
|  | L-Aspartate | **10.0** |
|  | L-Cysteine | **2.4** |
|  | L-Glutamine | **1.1** |
|  | L-Glutamate | **8.8** |
|  | L-Histidine | **0.0** |
|  | L-Isoleucine | **0.0** |
|  | L-Leucine | **0.0** |
|  | L-Lysine | **0.4** |
|  | L-Methionine | **0.0** |
|  | L-Phenylalanine | **0.0** |
|  | L-Proline | **0.0** |
|  | L-Serine | **0.0** |
|  | L-Threonine | **0.0** |
|  | L-Tryptophan | **0.0** |
|  | L-Tyrosin | **0.9** |
|  | L-Valine | **0.0** |
|  |  |  |
| **Fatty acids, carboxylic acids, keto acids** | | |
|  | Acetate | **7.9** |
|  | 3-Methylbutyrate | **0.0** |
|  | Butyrate | **1.8** |
|  | Capric acid | **0.0** |
|  | Caprylic acid | **0.0** |
|  | DL-Lactate | **0.0** |
|  | n-Caproic acid | **0.0** |
|  | Propionate | **3.9** |
|  | Formate | **0.0** |
|  | 6-Phosphogluconate | **0.0** |
|  | Citrate | **0.0** |
|  | DL-Malate | **12.0** |
|  | Fumarate | **9.4** |
|  | Glycolate | **0.0** |
|  | Glyoxylate | **2.1** |
|  | Isobutyrate | **3.8** |
|  | Malonate | **2.3** |
|  | Oxalate | **0.0** |
|  | Oxaloacetate | **0.0** |
|  | Succinate | **11.7** |
|  | alpha-Ketoglutarate | **9.2** |
|  | Levulinate | **1.0** |
|  | Phosphoenolpyruvate | **31.5** |
|  | Pyruvate | **21.0** |
|  |  |  |
| **Aldehydes** | |  |
|  | Acetaldehyde | **3.9** |
|  | Butyraldehyde | **0.0** |
|  | Formaldehyde | **0.0** |
|  | Glyoxal | **0.0** |
|  | Propionaldehyde | **0.0** |
|  |  |  |
| **Ketones** | |  |
|  | Aceton | **0.1** |
|  | 1,3-Dihydroxyaceton | **0.0** |
|  | 2-Butanone | **0.0** |
|  | Methylglyoxal | **0.0** |
|  |  |  |
| **Amines** | |  |
|  | Choline | **0.0** |
|  | Glycine betaine | **0.8** |
|  | L-Carnitine | **0.0** |
|  | Methylamine | **0.0** |
|  | Putrescine | **0.0** |
|  | Spermidine | **0.0** |
|  |  |  |
| **Nucleotides** | |  |
|  | Adenine | **0.0** |
|  | Cytosine | **0.0** |
|  | Guanin | **0.0** |
|  | Thymine | **0.0** |
|  |  |  |
| **Aromatic substances** | |  |
|  | Phenylacetate | **3.2** |
|  | Phenol | **0.0** |
|  | Benzoic acid | **0.0** |
|  |  |  |
| **Esters (experiments with 15 mg L^-1^ substrate concentration)** | | |
|  | Phenylacetate | **+^§§^** |
|  | Ethylpyruvate | **-^§§^** |
|  | α-terpinylacetate | **-^§§^** |
|  | Isoamylacetate | **-^§§^** |
|  | Methylacetate | **+^§§^** |
|  |  |  |
| **Humic substances and photooxidation products** | | |
|  | Humic Substances | **0.0** |
|  | Photooxidation products of humic substances ^§^ | **8.7** |
|  |  |  |
| **Bacteriological complex medium** | | |
|  | 10% NSY | **4.4** |
|  |  |  |
|  |  |  |
| ^%^ carbon content unknown, assimilation of substance was observed | | |
| ^§^ a carbon content of 22% of dry weight was determined | | |
| ^§§^ growth efficiency was not determined | | |
|  |  |  |
