# Supplementary material for: The Passive Yet Successful Way of Planktonic Life: Genomic and Experimental Analysis of the Ecology of a Free-Living Polynucleobacter Population
Source: PLoS One. 2012 Mar 20;7(3):e32772. doi: 10.1371/journal.pone.0032772 (PMC3308952; doi:10.1371/journal.pone.0032772)
Supplement: Table S4 — Loci used for the multilocus sequence analysis of the eleven F10 lineage strains isolated from Pond-1 (compare Fig. 5). The sequences of Pnuc_1240 were not considered for the calculation of the tree shown in Fig. 5. (DOCX) [file pone.0032772.s004.docx]

| **Gene/Spacer** | **Gene tag** | **Fragment length^§^ (nt)** | **# Polymorphic sites** | **Position in genome** |
| --- | --- | --- | --- | --- |
|  |  |  |  |  |
| gyrB | Pnuc_0003 | 873 | - | 2851-5349 |
| 16S-23S ITS^$^ | - | 506 | - | 38362-38868 |
| icd2 | Pnuc_0366 | 878 | 8 | 360802-363036 |
| hemF | Pnuc_0616 | 742 | - | 604744-605676 |
| ilvD | Pnuc_0884 | 428 | - | 876398-878146 |
| - | Pnuc_1095 | 1676 | 7 | 1102872-1134161 |
| - | Pnuc_1239 | 298 | - | 1302926-1304254 |
| - | Pnuc_1240 | - | insertion of IS | 1304359-1305312 |
| - | Pnuc_1241 | 264 | - | 1305340-1306224 |
| glnA | Pnuc_1255 | 615 | - | 1320425-1321840 |
| iscS | Pnuc_1493 | 730 | - | 1568270-1569529 |
| aspS | Pnuc_1892 | 995 | - | 1967417-1969216 |
| - | Pnuc_2073 | 930 | - | 2140109-2142313 |
|  |  |  |  |  |
|  |  |  |  |  |
| ^$^ intergenic transcribed spacer (ITS) including tRNA-Ile and tRNA-Ala genes | | | | |
| ^§^ fragments used for multi locus sequence analysis | | | |  |
