# Supplementary material for: The Passive Yet Successful Way of Planktonic Life: Genomic and Experimental Analysis of the Ecology of a Free-Living Polynucleobacter Population
Source: PLoS One. 2012 Mar 20;7(3):e32772. doi: 10.1371/journal.pone.0032772 (PMC3308952; doi:10.1371/journal.pone.0032772)
Supplement: Table S5 — Loci used for the multilocus sequence analysis shown in Fig. 8. (DOCX) [file pone.0032772.s005.docx]

| **Gene** | **Encoded product** | **Locus tag^$^** | **Gene length^$^ (nt)** | **Position in genome^§^** |
| --- | --- | --- | --- | --- |
|  |  |  |  |  |
| dnaA | Chromosomal replication initiator protein | Pnuc_0001 | 1428 | 33 |
| gyrB | DNA gyrase subunit B | Pnuc_0003 | 2499 | 2851 |
| atpD | F1 sector of membrane-bound ATP synthase | Pnuc_0024 | 1542 | 21873 |
| rpoB | DNA-directed RNA polymerase subunitB | Pnuc_0046 | 4101 | 47471 |
| gltA | Citrate synthase | Pnuc_0763 | 1314 | 763471 |
| glnA | Glutamine synthetase | Pnuc_1255 | 1416 | 1320425 |
| lpxB | Lipid A disaccharide synthetase | Pnuc_1438 | 1206 | 1508834 |
| recA | Recombination protein RecA | Pnuc_1833 | 1092 | 1913680 |
|  |  |  |  |  |
|  |  |  |  |  |
| ^$^ genome annotation of QLW-P1DMWA-1 | |  |  |  |
| ^§^ genome sequence of QLW-P1DMWA-1 (total length 2159490 nt) | | |  |  |
