# Supplementary material for: The Passive Yet Successful Way of Planktonic Life: Genomic and Experimental Analysis of the Ecology of a Free-Living Polynucleobacter Population
Source: PLoS One. 2012 Mar 20;7(3):e32772. doi: 10.1371/journal.pone.0032772 (PMC3308952; doi:10.1371/journal.pone.0032772)
Supplement: Table S6 — Genomes and strains considered for the multilocus sequence analysis presented in Fig. 8. (DOCX) [file pone.0032772.s006.docx]

| **Species (synonym)** | **Strain** | **Family** | **Accession numbers** |
| --- | --- | --- | --- |
|  |  |  |  |
| *Polynucleobacter necessarius* ssp. *asymbioticus* | QLW-P1DMWA-1^T^ | *Burkholderiaceae* | NC_009379 |
| *Burkholderia cepacia* | 383 (= R18194) | *Burkholderiaceae* | NC_007509 - NC_007511 |
| *Burkholderia multivorans* | ATCC 17616 | *Burkholderiaceae* | NC_010801, NC_010802, NC_010804, NC_010805 |
| *Burkholderia xenovorans* (*B. fungorum)* | LB400 | *Burkholderiaceae* | NC_007951 - NC_007953 |
| *Ralstonia pickettii* | 12J | *Burkholderiaceae* | NC_010678, NC_010682, NC_010683 |
| *Ralstonia solanacearum* | GMI1000 | *Burkholderiaceae* | NC_003295, NC_003296 |
| *Cupriavidus necator* (*Ralstonia eutropha*) | H16 | *Burkholderiaceae* | NC_008313, NC_008314 |
| *Cupriavidus* cf. *necator* (*Ralstonia eutropha*) | JMP134 | *Burkholderiaceae* | NC_007336, NC_007347, NC_007348 |
| *Cupriavidus taiwanensis* | LMG19424 | *Burkholderiaceae* | NC_010528 - NC_010530 |
| *Cupriavidus metallidurans* | CH34 | *Burkholderiaceae* | NC_007971 - NC_007974 |
| *Comamonas testosteroni* | KF-1 | *Comamonadaceae* | NZ_AAUJ00000000 |
|  |  |  |  |
