# Supplementary material for: The Passive Yet Successful Way of Planktonic Life: Genomic and Experimental Analysis of the Ecology of a Free-Living Polynucleobacter Population
Source: PLoS One. 2012 Mar 20;7(3):e32772. doi: 10.1371/journal.pone.0032772 (PMC3308952; doi:10.1371/journal.pone.0032772)
Supplement: Table S7 — Bootstrap values obtained in phylogenetic reconstructions using the concatenated amino acid (AA) or nucleotide sequences (nt) of eight housekeeping genes (Fig. 8). For comparison, phylogenetic reconstructions solely based on the 16S rRNA gene sequences of the same set of organisms were performed (tree not shown). Note the strong support for node E placing the Polynucleobacter strain next to the Ralstonia/Cupriavidus clade. (DOCX) [file pone.0032772.s007.docx]

| **Node** | **Eight concatenated genes (AA)** | | |  | **Eight concatenated genes (nt)** | | |  | **16S rRNA gene** | | |
| --- | --- | --- | --- | --- | --- | --- | --- | --- | --- | --- | --- |
|  | (NJ^&^) | (MP) | (ML^$^) |  | (NJ^§^) | (MP) | (ML^§^) |  | (NJ^§^) | (MP) | (ML^§^) |
|  |  |  |  |  |  |  |  |  |  |  |  |
| A | 100 | 99 | 100 |  | 100 | 100 | 100 |  | 40 | - | - |
| B | 100 | 97 | 99 |  | 100 | 99 | 99 |  | 44 | - | - |
| C | 100 | 100 | 100 |  | 100 | 100 | 100 |  | 79 | 38 | 33 |
| D | 100 | 100 | 100 |  | 100 | 100 | 100 |  | 95 | - | 40 |
| E | 100 | 100 | 100 |  | 100 | 100 | 100 |  | 100 | 100 | 98 |
| F | 66 | - | 67 |  | - | - | 60 |  | 100 | - | 99 |
| G | 100 | 100 | 100 |  | 100 | 100 | 100 |  | 100 | 99 | 98 |
| H | 100 | 100 | 100 |  | 100 | 100 | 100 |  | 100 | 95 | 46 |
|  |  |  |  |  |  |  |  |  |  |  |  |
| ^§^ using the best model (GRT+I+G) as identified by MEGA5 for ML, gamma distribution with 5 categories | | | | | | | | | | |  |
| ^&^ gamma distribution, 5 categories, pair-wise gap deletion | | | | | |  |  |  |  |  |  |
| ^$^ model rtREV+G+I+F | | |  |  |  |  |  |  |  |  |  |
