# Supplementary material for: The Passive Yet Successful Way of Planktonic Life: Genomic and Experimental Analysis of the Ecology of a Free-Living Polynucleobacter Population
Source: PLoS One. 2012 Mar 20;7(3):e32772. doi: 10.1371/journal.pone.0032772 (PMC3308952; doi:10.1371/journal.pone.0032772)
Supplement: Text S1 — Lack of genes well known from other Burkholderiaceae bacteria. (DOCX) [file pone.0032772.s008.docx]

The genome of *Polynucleobacter necessarius* ssp. *asymbioticus* strain QLW-P1DMWA-1 lacks any genes for flagella-driven motility and chemotaxis, confirming the previously reported phenotypic lack of motility (Hahn et al., 2009). The genome also encodes no genes for harvesting light energy, such as genes for anoxygenic photosynthesis (Yurkov & Beatty, 1998) or proteorhodopsin genes (Beja et al., 2000). Furthermore, genes known from related species – such as genes for oxidizing molecular hydrogen (*Cupriavidus necator* H16, synonym *Ralstonia eutropha*, (Pohlmann et al., 2006); *C. metallidurans* CH34, (Janssen et al., 2010)), genes potentially involved in oxidization of carbon monoxide (e,g., *C. metallidurans*), genes for cleavage of aromatic compounds (e.g., *C. necator* JMP134, (Lykidis et al., 2010)) genes for nitrate respiration (e.g., *C. necator*), genes for nitrogen fixation (*C. taiwanense* LMG19424, (Amadou et al., 2008)), as well as genes for CO_2_ fixation (*C. necator*) – are lacking. Furthermore, the genome seems to lack any ABC transporters for carbohydrates, as well as a phosphotransferase system (PTS). By contrast, most genomes of *Cupriavidus* and *Ralstonia* strains encode putative transporters for carbohydrates.

According to CRISPRFinder (Grissa et al., 2007) the genome of QLW-P1DMWA-1 lacks any CRISPR structures (Clustered Regularly Interspaced Short Palindromic Repeats). However, with the exception of three strains, such sequences are also largely absent in the genomes of other *Burkholderiacea* species.

**References not listed in the main reference list**

**Amadou C, Pascal G, Mangenot S, Glew M, Bontemps C, et al. (2008)** Genome sequence of the beta-rhizobium *Cupriavidus taiwanensis* and comparative genomics of rhizobia. Genome Res 18: 1472-1483.

**Beja O, Aravind L, Koonin EV, Suzuki MT, Hadd A, et al. (2000)** Bacterial rhodopsin: Evidence for a new type of phototrophy in the sea. Science 289: 1902–1904.

**Grissa I, Vergnaud G, Pourcel C (2007)** CRISPRFinder: a web tool to identify clustered regularly interspaced short palindromic repeats. Nucleic Acids Res 35: W52-W57.

**Hahn MW, Lang E, Brandt U, Wu QL, Scheuerl T (2009)** Emended description of the genus *Polynucleobacter* and the species *P. necessarius* and proposal of two subspecies, *P. necessarius* subspecies *necessarius* subsp. nov. and *P. necessarius* subsp. *asymbioticus* subsp. nov. Int J Syst Evol Microbiol 59: 2002-2009.

**Lykidis A, Pérez-Pantoja D, Ledger T, Mavromatis K, Anderson IJ, et al. (2010)** The complete multipartite genome sequence of *Cupriavidus necator* JMP134, a versatile pollutant degrader. PLoS ONE 5: e9729.

**Pohlmann A, Fricke W, Reinecke F, Kusian B, Liesegang H, et al. (2006)** Genome sequence of the bioplastic-producing Knallgas bacterium *Ralstonia eutropha* H16. Nature Biotechnol 24: 1257–1262.

**Yurkov VV, Beatty JT (1998)** Aerobic anoxygenic phototrophic bacteria. Microbiol Mol Biol Rev 62: 695–724.
