# Supplementary material for: The Passive Yet Successful Way of Planktonic Life: Genomic and Experimental Analysis of the Ecology of a Free-Living Polynucleobacter Population
Source: PLoS One. 2012 Mar 20;7(3):e32772. doi: 10.1371/journal.pone.0032772 (PMC3308952; doi:10.1371/journal.pone.0032772)
Supplement: Text S2 — Search for genes involved in adaptations to a planktonic lifestyle. (DOCX) [file pone.0032772.s009.docx]

The comparison of the QLW-P1DMWA-1 genome with all genomes of *Cupriavidus* (n = 4) and *Ralstonia* strains (n = 5) available at the time of analysis in the IMG system identified 38 clusters of orthologous groups (COGs), which are overrepresented by gene numbers (7 COGs representing 23 genes) or exclusively present in the *Polynucleobacter* genome (31 COGs with 36 genes). The majority of COGs belong to the categories S (function unknown, 17 COGs, 17 genes), M (cell envelope biogenesis, outer membrane, 4 COGs, 10 genes), R (general function prediction only, 4 COGs, 8 genes), and C (energy production and conversion, 4 COGs, 4 genes). Among the 59 genes representing the 38 COGs an overrepresentation of transmembrane proteins (42.4% versus 25.1% in entire genome), proteins with signal peptides (33.9% versus 22.5% in entire genome), and genes with either low (≤ 40%) or high (≥ 50%) GC content (in total 27.1% versus 9.3% of genes in genome) was observed. The latter may indicate a relatively recent horizontal gene transfer of at least some of the genes with rather atypical GC content. A few of the genes found in this analysis may represent prophage genes. None of the genes seem to indicate an advantageous adaptation to a planktonic lifestyle.

One interesting gene regarding adaptation to planktonic life was, however, overlooked by the COG as well as by a similar protein families (Pfam) analysis. QLW-P1DMWA-1 encodes an open reading frame (Pnuc_1095) of a length (31290 bp) only rarely found among other bacteria (Reva & Tümmler, 2008). Among the 580 sequenced prokaryotic genomes only 14% (representing 47 taxa) encoded giant genes (> 20 kb). Like other giant genes investigated by Reva & Tümmler, Pnuc_1095 encodes a putative protein, which is acidic, threonine-rich, lacks cystein and is characterized by amino acid repeats, however Pnuc_1095 shares only low sequence identity with other giant genes. Analysis with the service PRED-TMMB (<http://biophysics.biol.uoa.gr/PRED-TMBB/>) indicates that Pnuc_1095 encodes an outer membrane protein. This is also indicated by the putative presence of a C-terminal outer membrane autotransporter barrel. Comparative analysis of the gene sequence yielded no reliable hints on the function of the putatively encoded 10429 amino acids protein. Most of the giant genes identified so far were present in environmental bacteria, including several planktonic bacteria. For instance, *Candidatus* Pelagibacter ubique, some *Synechococcus* strains and a few *Prochlorococcus* strains encoded such giant genes (Reva & Tümmler, 2008; Scanlan et al., 2009). In one *Synechococcus* strain the encoded protein enables a flagella-independent motility. In most of the other cases, it is assumed that the giant genes encode surface proteins, which shield the cell against negative environmental influences, which may result in resistance to viral infection or protection against grazing (Scanlan et al., 2009).

**Reference not listed in the main reference list**

**Reva O, Tümmler B (2008)** Think big – giant genes in bacteria. Environ Microbiol. 10: 768-77.
