# Supplementary material for: The Passive Yet Successful Way of Planktonic Life: Genomic and Experimental Analysis of the Ecology of a Free-Living Polynucleobacter Population
Source: PLoS One. 2012 Mar 20;7(3):e32772. doi: 10.1371/journal.pone.0032772 (PMC3308952; doi:10.1371/journal.pone.0032772)
Supplement: Text S3 — Experiment on growth support of Polynucleobacter bacteria by algal exudates. (DOCX) [file pone.0032772.s010.docx]

An experiment on potential utilization of algal exudates as major substrate source for *Polynucleobacter* bacteria originating from Pond-1 was conducted under laboratory conditions as described in the Materials and Methods section. A 0.8 µm-filtered (predator-free) water sample from Pond-1 was used for this experiment. On the day of sampling, PnecC bacteria comprised 33.5% of total bacterial numbers, and the F10 lineage population comprised 41.9 % of PnecC numbers in Pond-1 (Fig. 2). Relative abundance of PnecC bacteria changed slightly during the preparation of the experiment (Fig. 12). Subsamples of the filtered water sample were mixed with algal cultures and incubated in the light. All three algal species (representing species present in Pond-1) increased in numbers during the course of the experiment but peaked after different incubation periods. *Dinobryon sp.* showed the strongest growth, with increases in cell numbers from initially 5.4 x 10^3^ cells mL^-1^ (t = 0 d) to 104.0 x 10^3^ cells mL^-1^ (t = 8 d). After the peak, numbers fell slightly to 84.7 x 10^3^ cells mL^-1^ at the end of the experiment (t = 15 d). *Synura* sp. numbers increased from initially 45.6 x 10^3^ to 70.6 x 10^3^ cells mL^-1^ (t = 6 d) and fell slightly afterwards to 67.4 x 10^3^ cells mL^-1^ (t = 15 d). *Ochromonas* sp. numbers increased over the entire duration of the experiment from initially 13.5 x 10^3^ to 27.3 x 10^3^ cells mL^-1^(t = 15 d). Total bacterial numbers changed little in the *Synura* and *Dinobryon* treatments during the experiment but increased in the *Ochromonas* treatment from initially 4.6 x 10^6^ cells mL^-1^ to 9.0 x 10^6^ cells mL^-1^ (t = 4 d) and decreased thereafter to 3.0 x 10^6^ cells mL^-1^ until the end of the experiment. In all three algal treatments the final (t = 15 d) total bacterial numbers were lower than the initial numbers (57 %, 81 %, and 66 % of initial cell numbers in *Dinobryon*, *Synura*, and *Ochromonas* treatments, respectively) while the numbers increased in the algae-free control treatment (136 % of initial numbers). The decrease of bacterial numbers in the *Dinobryon* and the *Ochromonas* treatment but not in the *Synura* treatment could have partially been caused by potential mixotrophic capabilities of the two former algae. By contrast, *Synura* is completely lacking any phagotrophic capabilities. Apart from the control treatment, the relative abundance of *Polynucleobacter* bacteria did not increase significantly during the experiment (Fig. 12). In contrast to the control treatment, relative PnecC numbers steadily decreased in the *Dinobryon* and *Synura* treatments. Solely the *Ochromonas* treatment showed a more or less unchanged proportion of PnecC bacteria.
